# Supplementary material for: Genetic analyses implicate complex links between adult testosterone levels and health and disease
Source: Commun Med (Lond). 2023 Jan 18;3:4. doi: 10.1038/s43856-022-00226-0 (PMC9849476; doi:10.1038/s43856-022-00226-0)
Supplement: Supplementary file 14 — Supplementary Information [file 43856_2022_226_MOESM14_ESM.pdf]

Supplementary Figures and Legends

Supplementary Figure 1. Results from tissue enrichment analyses from MAGMA implemented in FUMA

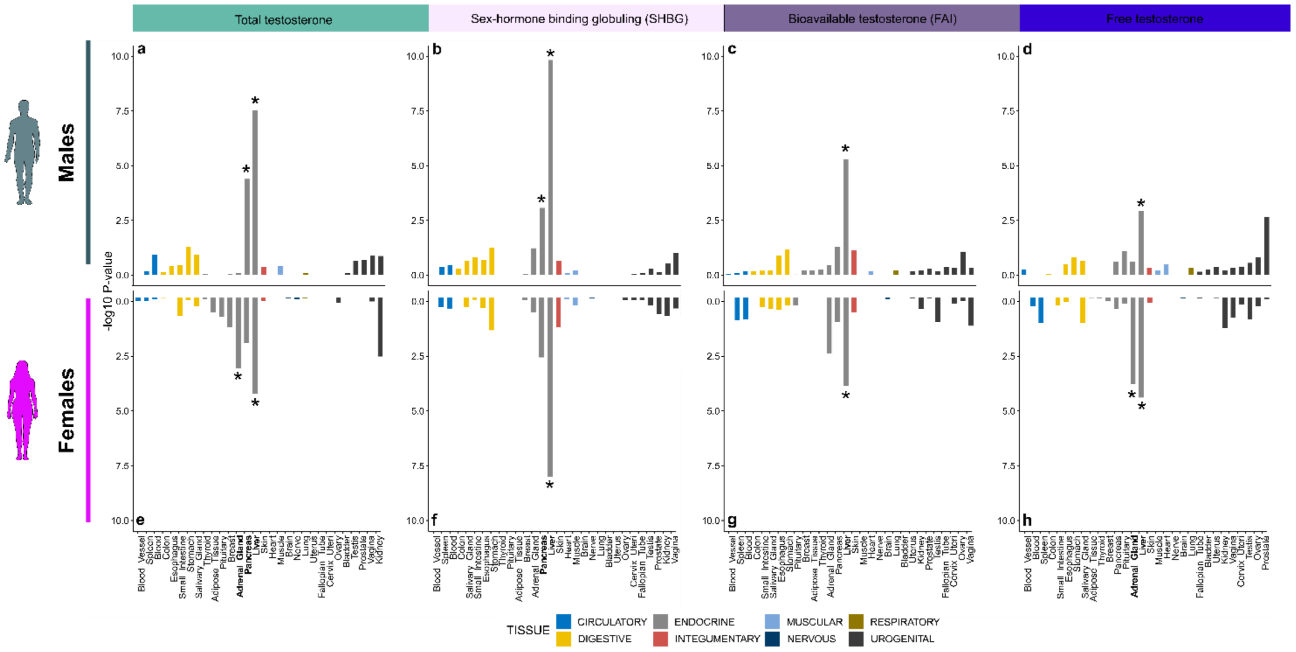

Panels **a-d** show tissue enrichment in males, and **e-h** in females. Green = total testosterone, white lilac = SHBG, grey = free androgen index (FAI), blue = free testosterone. The GTEx tissues shown in figure have been group based on tissue types, with code for tissue colors available in the figure. Based on the UK Biobank GWAS, a large proportion of the genes residing in the GWAS loci are preferably expressed in the liver. This applies to all studied traits. Interestingly, we simultaneously detected no evidence for the GWAS loci being enriched for genes expressed in the testis or the ovaries, the tissues responsible for the bulk of T production. Potentially, this can be a sign that at population level, individual differences in T metabolism may have larger effects on adult serum T levels than genetic differences in the tissues responsible for T production. Especially for males it seems thus possible that T production may not be the rate-limiting step for T levels. However, the situation may be slightly different in females, where adrenal gland is a major site for T production, and many genes in the loci associated with total and free T in females appeared preferably expressed in this tissue. Underscoring the close relationship between metabolism and T levels, pancreas also shows up as a tissue enriched for the GWAS signals for total T and SHBG in males.

Supplementary Figure 2. Visualisation of results from KEGG pathway analyses in FUMA

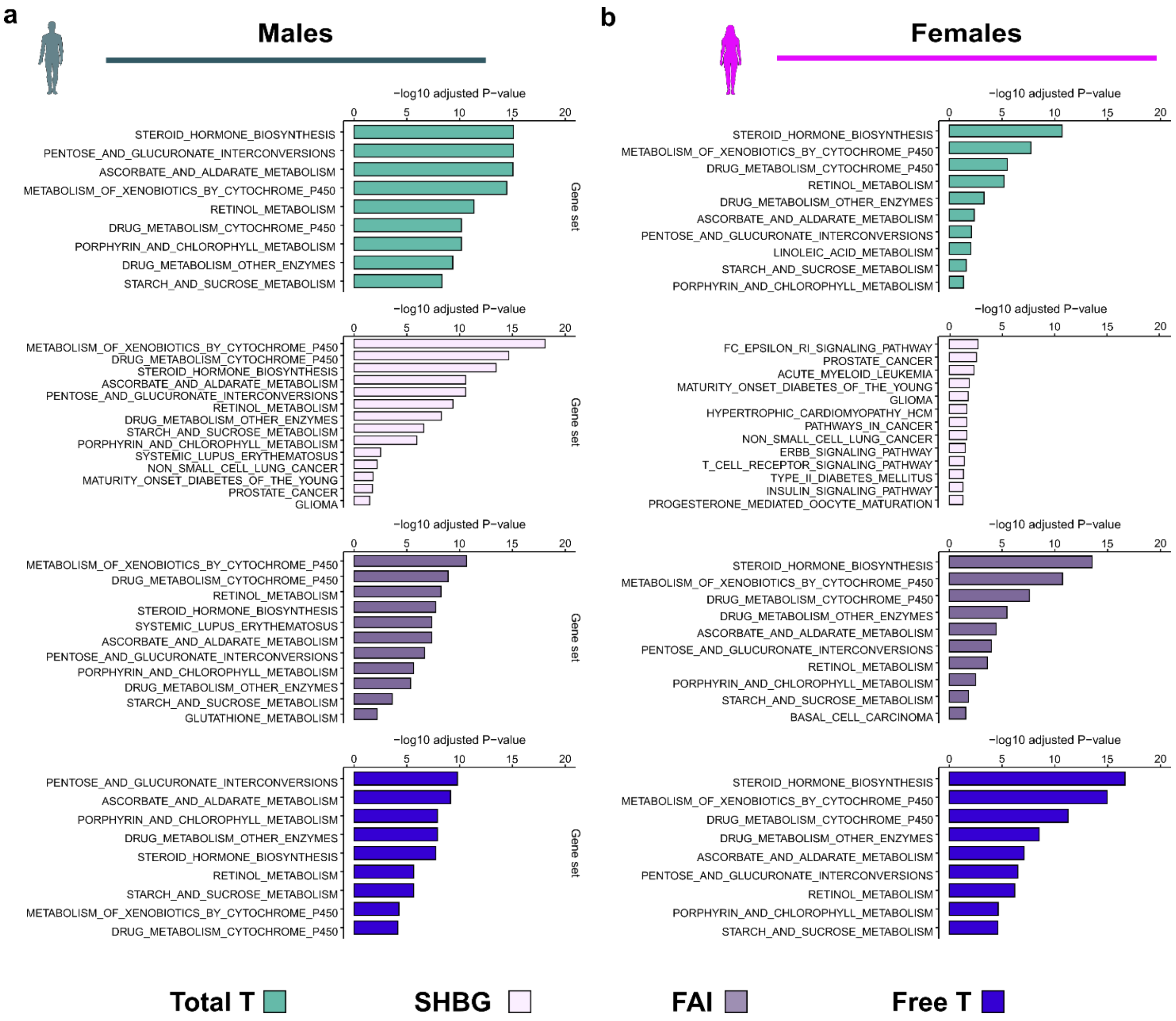

The figure illustrates Kyoto Encyclopedia of Genes and Genomes (KEGG) pathways showing enrichment for the genes residing in the GWAS loci. Panel **a** shows the results for males, and **b** for females. Green = enriched pathways for total testosterone, white lilac = SHBG, grey = free androgen index (FAI), blue = free testosterone. These results are based on statistically significant enrichment on KEGG pathways, after adjusting for multiple testing in FUMA (Supplementary Table 10). The GWAS loci show clear enrichment for genes that affect steroid hormone biosynthesis, various metabolic pathways and metabolite excretion. Overall these results highlight that the genetic variation detected in the GWASs relates to many molecular pathways that are known to be crucial for T and SHBG processing and regulation. Importantly, these results also underscore that no single pathway dominates T regulation, but that genetic regulation of T levels in the human body likely results from simultaneous and combined action of many molecular processes. Notably, the results support the observations from the tissue enrichment analyses, emphasizing the significance of T metabolism (for which the major site of action is

liver) to T levels, likely via specific and unspecific mechanisms. T=Testosterone, SHBG = Sex-hormone binding globulin, FAI = Free androgen index, Free T = calculated free testosterone.

Supplementary Figure 3. Correlation between testosterone levels ~5 years apart for a subset of UK Biobank participants

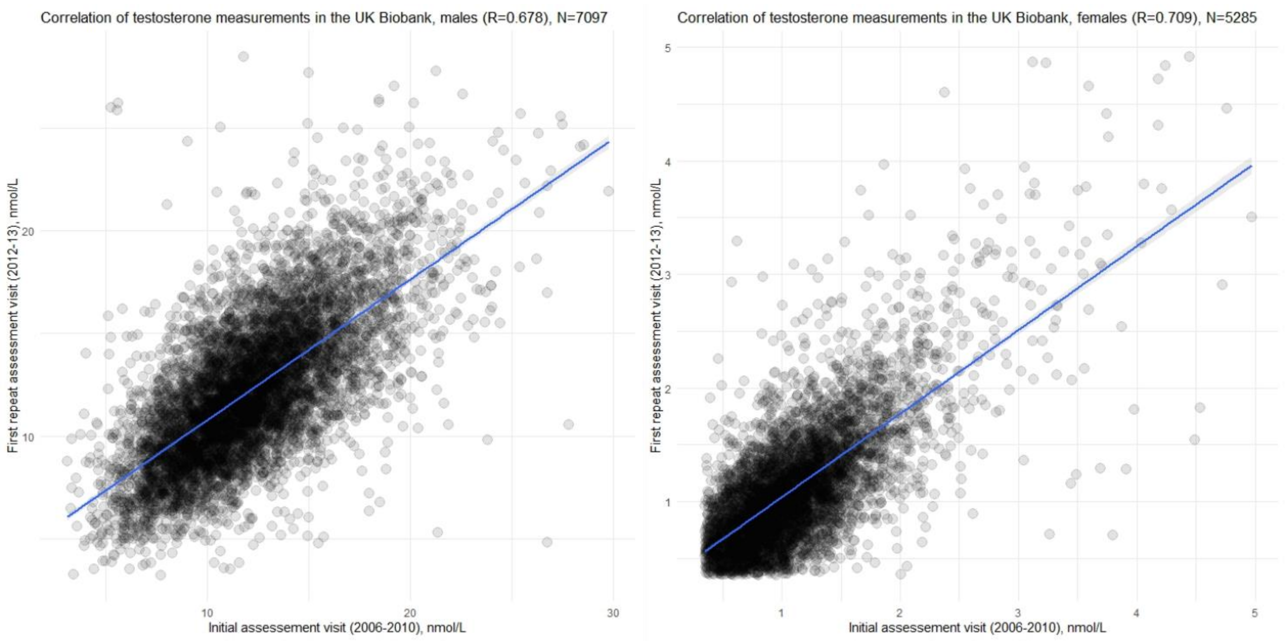

For both men (N=7,097) and women (N=5,285), testosterone (T) measurements from roughly five years apart are highly correlated (R=0.678 in men, R=0.709 in women) in the UK Biobank. The grey circles indicate individual measurements at time point 1 (original visit, x-axis) vs. time point 2 (repeat assessment, y-axis). The high correlations of T measurements between the two distinct time points provide strong evidence that the immunohistochemical method used reliably captures population-level variability in T levels. These correlations support also the concept that there exists a fairly stable individual-level baseline for T, known to be largely heritable in basis, reflecting an individual's life-long T exposure. Further supporting such a baseline for T levels, measurement at the first visit, several years prior the second, predicts T level at the second visit considerably better than for instance BMI from the current visit (Supplementary Figure 10). For visual purposes, the individuals with a likely medical condition leading to extremely low or high testosterone (<3 in men and >5 in women) were removed from the plots.

Supplementary Figure 4. Genetic correlation results between the studied testosterone traits

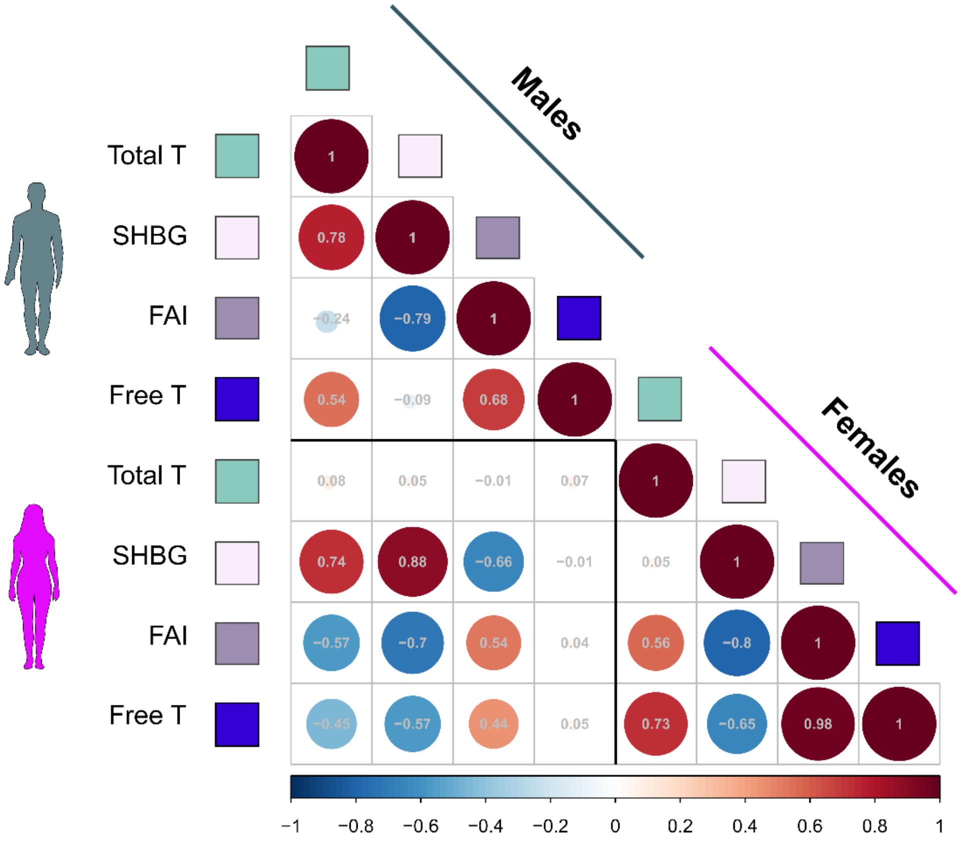

The figure shows genetic correlations based on LDSC analyses for males (area next to dark green symbol), females (pink) and over both sexes (bottom left corner surrounded by black lines in the correlation grid). Green = total testosterone, white lilac = SHBG, grey = free androgen index (FAI), blue = free testosterone. In line with recent findings, we observed only a very weak genetic correlation for total T levels between males and females (r_g=0.08, *p*=0.063). Similarly, free T showed low genetic correlation estimate between the sexes (r_g=0.05, *p*=0.102). This supports the concept that uniquely among complex traits, T levels are determined by distinct heritable factors in males and females, echoing results from earlier twin studies (20). In contrast, the genetic correlation for males and females SHBG was high (r_g=0.88, *p*=9.7e-197), and for FAI intermediate (r_g=0.57, *p*=5.8e-26), suggesting that similar genetic factors contribute to these traits in both sexes. Moreover, consistent with epidemiological observations that total T and SHBG levels are correlated in males, these traits showed a robust positive genetic correlation (r_g=0.78, *p*=1.0e-127). This observation supports the concept that under normal physiological conditions, based on homeostatic feedback SHBG levels increase when T increases and vice versa. At the same time, under the premise of unbound T being biologically the most potent form of T, this suggests that some genetic variants affecting total T in men do not necessarily reflect increased T activity. Yet, for females such connection between

total T and SHBG did not exist (r_g=0.05, p=0.207), suggesting such feedback mechanism is not active in females, and that total T levels in females may be more closely related to T action than total T levels in males.

Supplementary Figure 5. Predictive ability of total T and free T PGSs for T levels in the UK Biobank

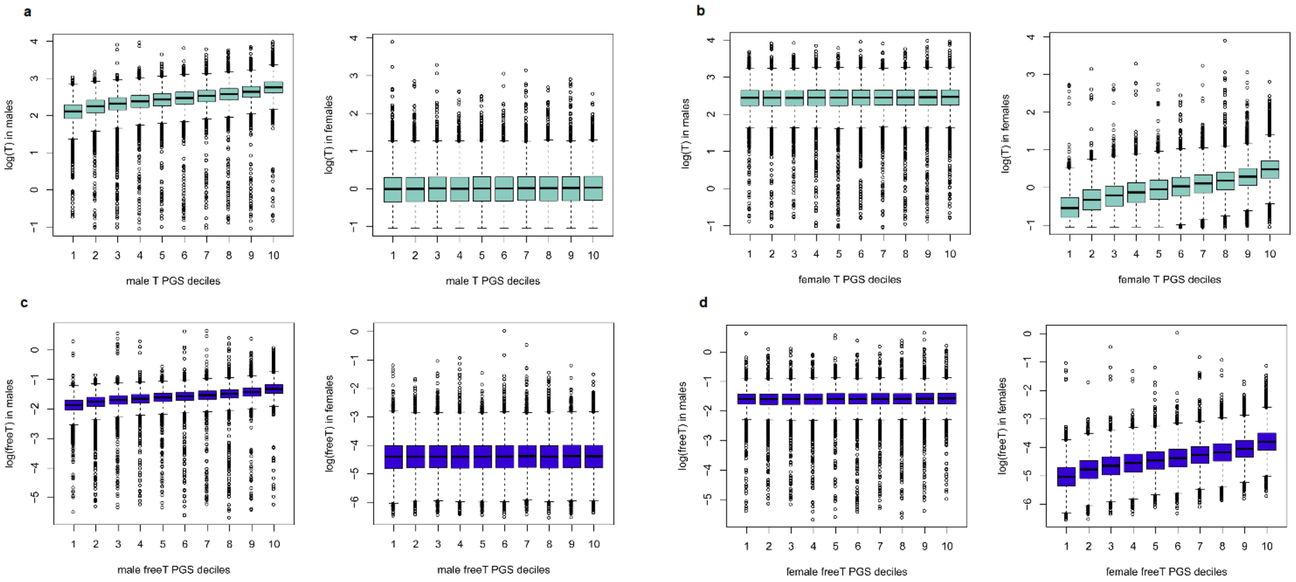

The figure shows how the sex-specific PGS correlates highly with T levels within the sex it was constructed, and has drastically limited predictive ability in the opposite sex. **a** Total T PGS vs T in men, **b** Total T PGS vs T in women, **c** Free T PGS in men, **d** Free T PGS in women. The box plots show median (black line), lower and upper quartiles (colored area of the box) for log T and free T per PGS decile (1=persons with T PGS in the lowest 10%, 10 = persons with T PGS higher than for 90% of the samples), and the error bars indicate 5% and 95% quantiles. Green boxplots = total testosterone values by PGS decile, blue = free testosterone values by PGS decile. Although the cross-sex comparisons in UK Biobank are performed using independent datasets (men vs. women, based on data from 159,110 unrelated men and 184,573 unrelated women with white British ancestry), please note that the training (PGS calculation) and the test (explained variance) datasets for the sex-specific PGSs include the same individuals from the UK Biobank, leading to inflated results.

Supplementary Figure 6. PGS associations in the FinnGen

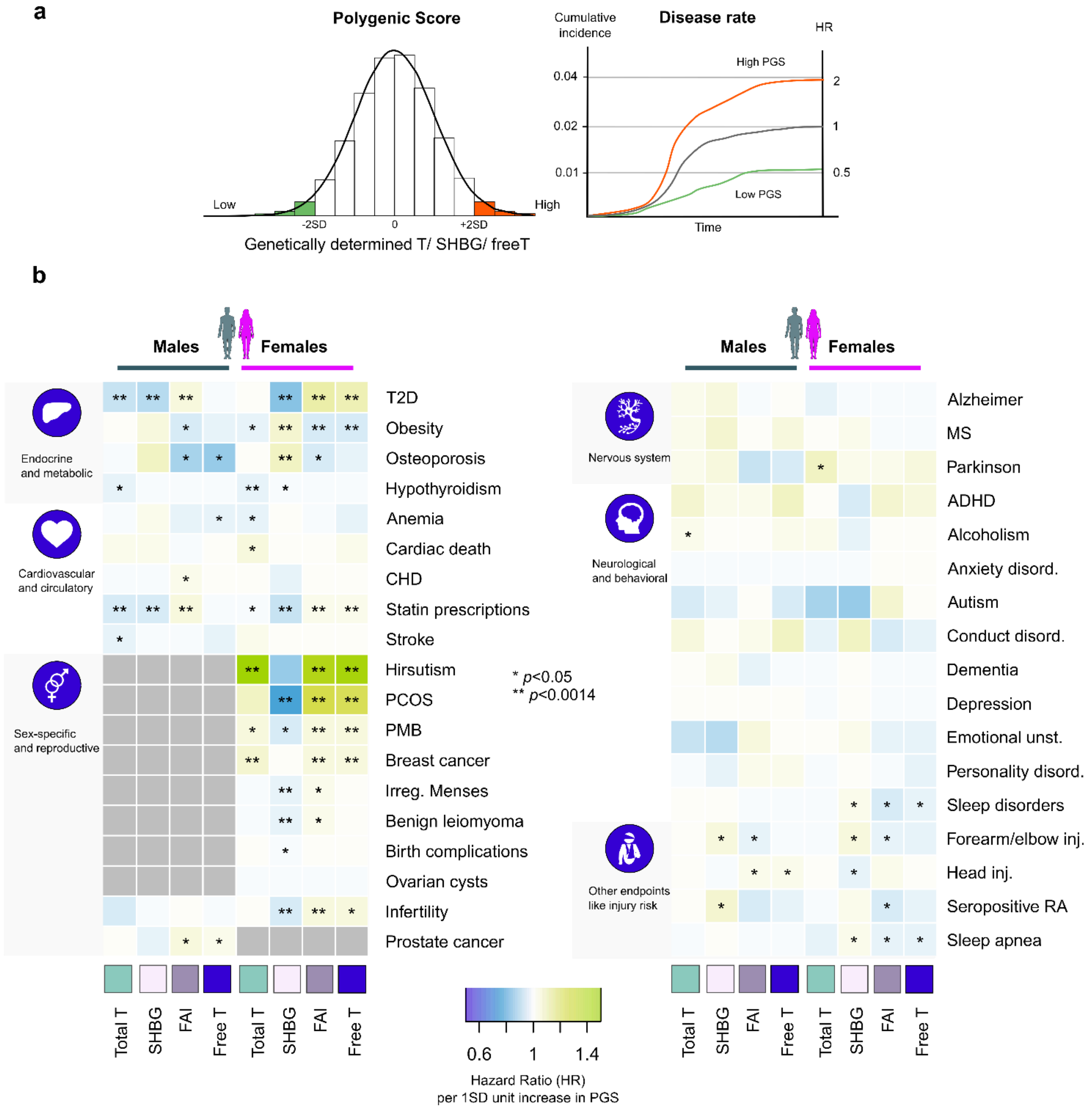

a Rationale of the PGS analyses. Calculation of the PGS in the FinnGen allows for ranking participants according to their genetic predisposition to higher T/ SHBG and free T levels. Then, the consequences of having either high/low genetically determined T can be estimated using the clinical information available from the FinnGen dataset. The figure on the right shows an example case where having a high PGS (2SD above population mean, orange) doubles the hazard ratio (HR) of getting diagnosed with a disease compared to those with an average PGS (grey line). The line green indicates those with low PGS (2SD below population mean) have only half of the risk of getting the diagnosis. **b** The heatmap illustrates hazard

ratios (HR) per 1SD increase in PGS for all studied traits in the sex-specific analyses. $p<0.0014$ corresponds to Bonferroni correction for 36 traits. Dark green symbol = males, pink = females. Green circles = total testosterone, white lilac = SHBG, grey = free androgen index, blue = free testosterone. Yellow shades = increased risk for endpoint, blue shades = decreased risk for endpoint. Grey box = endpoint not available for this sex. The data is based on 94,478 men and 122,986 women from FinnGen R5.

Supplementary Figure 7. Schematic illustration of the power analysis for FinnGen disease associations

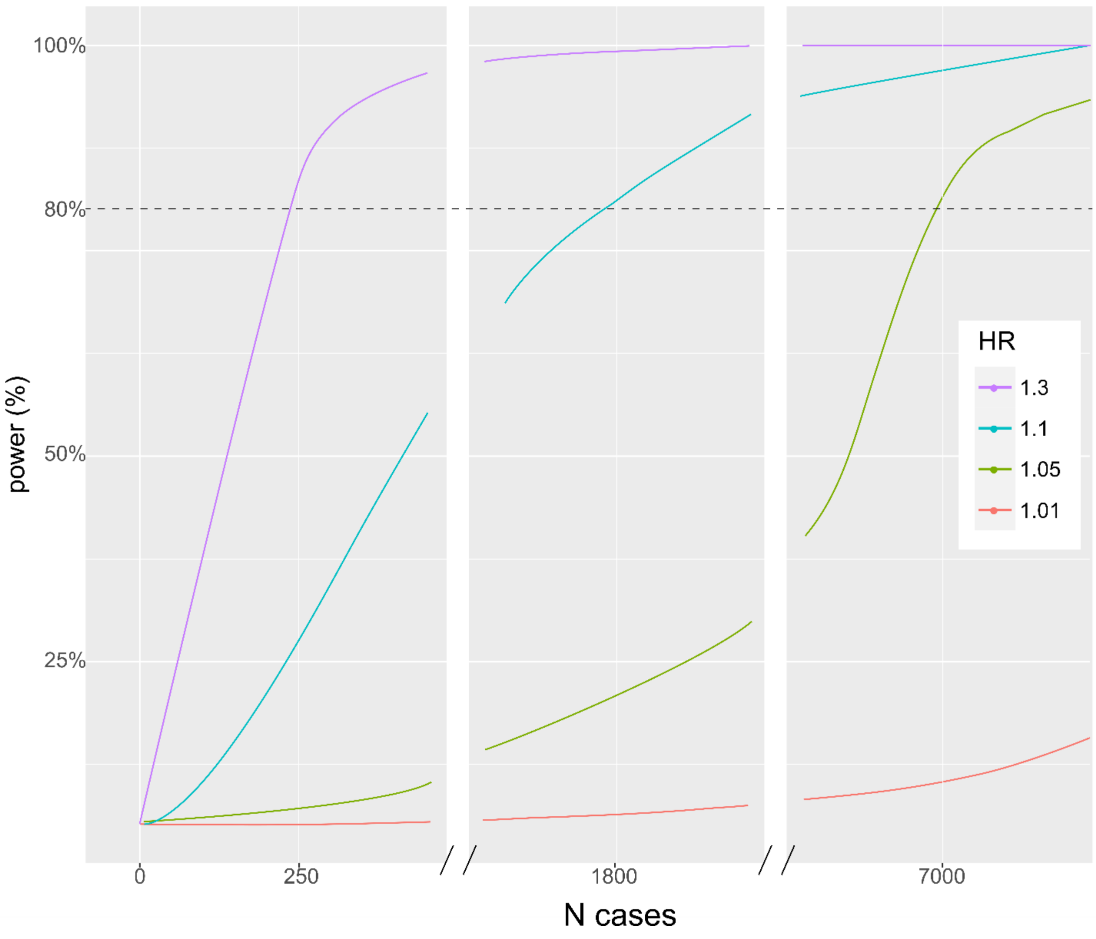

We estimate that even for the rarest phenotypes studied, given the selected *p*-value threshold (0.0014), we had full power to detect any large effects (HR>1.3) for the PGSs, and that we could reliably detect also subtler effects for most phenotypes. To exemplify this in practical terms, for rare diagnoses such as hirsutism and conduct disorder we had adequate power (>80%) to detect large effects (HR>1.3, purple line). We could detect at least intermediate effects (HR>1.1, blue line) for the vast majority of the disease endpoints (over 1800 cases, 49/64 of the studied sex-specific endpoints (64 = the count of the studied disease endpoints when a sex-shared endpoint is considered as two independent endpoints, e.g. both male and female statin use are added to total sum). For the most common phenotypes like T2D and depression (more than 7000 cases, representing roughly a third of all included endpoints (20/64), we had the power to catch even smaller effects (HR>1.05, green line). We did not have the power to detect very small effects per 1SD change in PGS (HR<1.01, red line), yet such effects would be likely considered negligible in terms of medical relevance. The HR refers to risk per 1SD increase in the PGS for the studied T trait (total T, SHBG, FAI or free T) based on the PGS analysis in FinnGen. The curves are based on the selected *p*-value threshold of 0.0014, corresponding for multiple testing correction for 36 traits, and on power

calculation for Cox proportional Hazards Regression with powerSurvEpi package in R. Phenotypes and their N are listed in Supplementary Data 7 for both sexes separately. Please note that the X-axis marking case number is not continuous.

Supplementary Figure 8. Comparison of unadjusted and SHBG-adjusted T PGS associations in FinnGen.

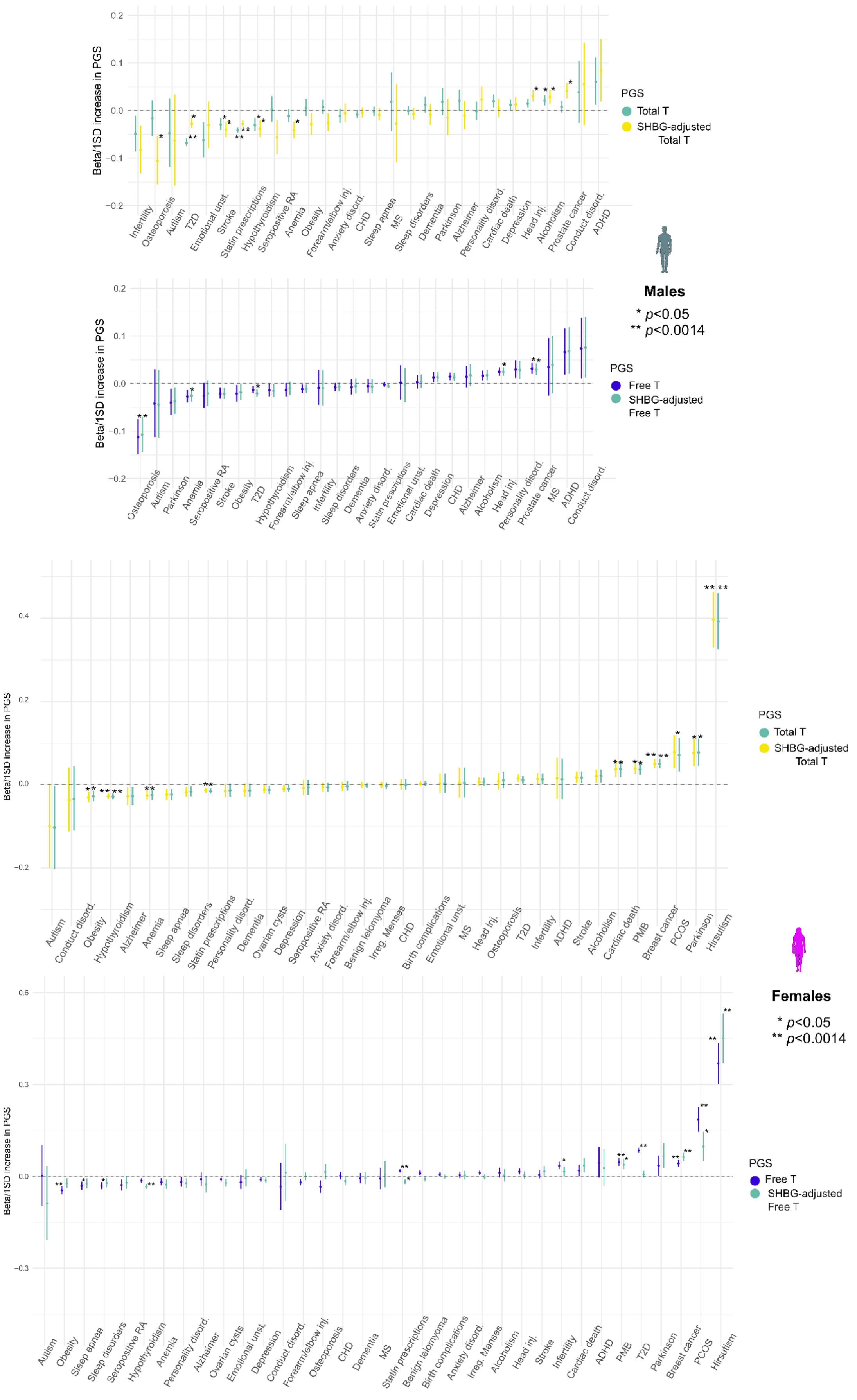

The figure shows disease risk per 1SD increase in PGS in a forest plot (beta and SE). Green = total T, yellow = SHBG-adjusted total T, blue = free T, light green = SHBG adjusted free T. The data is based on PGS association analyses comprising of 94,478 men and 122,986 women from FinnGen R5. For some endpoints, the effects between the unadjusted and the adjusted analysis appear different (e.g. only SHBG-adjusted total T showing nominally significant association to osteoporosis and prostate cancer in males). This suggests that the association of the unadjusted total T PGS to these endpoints suffers from the limitation that the PGS also reflects raised SHBG levels, which for these endpoints counteracts the effects of raised total T. This leads to at least two potential interpretations: first, this can be a sign of genetic pleiotropy, e.g. some genetic variants contributing to total T PGS affect both T and SHBG independently, or via another molecular pathway. Secondly, such a result may be a sign that a genetically set raise in SHBG leads to a biological compensation and raise in T levels (or vice versa), increased T binding by SHBG then counteracting the raise in T levels, and masking any associations that might result from direct (biologically available) T action.

Suggesting that both total T and SHBG in females likely contribute independently to PCOS, total T shows consistent association to PCOS both with and without SHBG adjustment, but free T association to PCOS is attenuated after SHBG adjustment. Notably, the statistically significant T associations to T2D disappear in both sexes (total T in men, free T in women) when taking into account the effect of SHBG in the analysis. For most other traits, the effects estimates remain highly similar between the unadjusted and adjusted analyses.

Supplementary Figure 9. Visualisation of genetic correlations and causality analyses between T, SHBG, FAI and free T and the studied disease endpoints in FinnGen

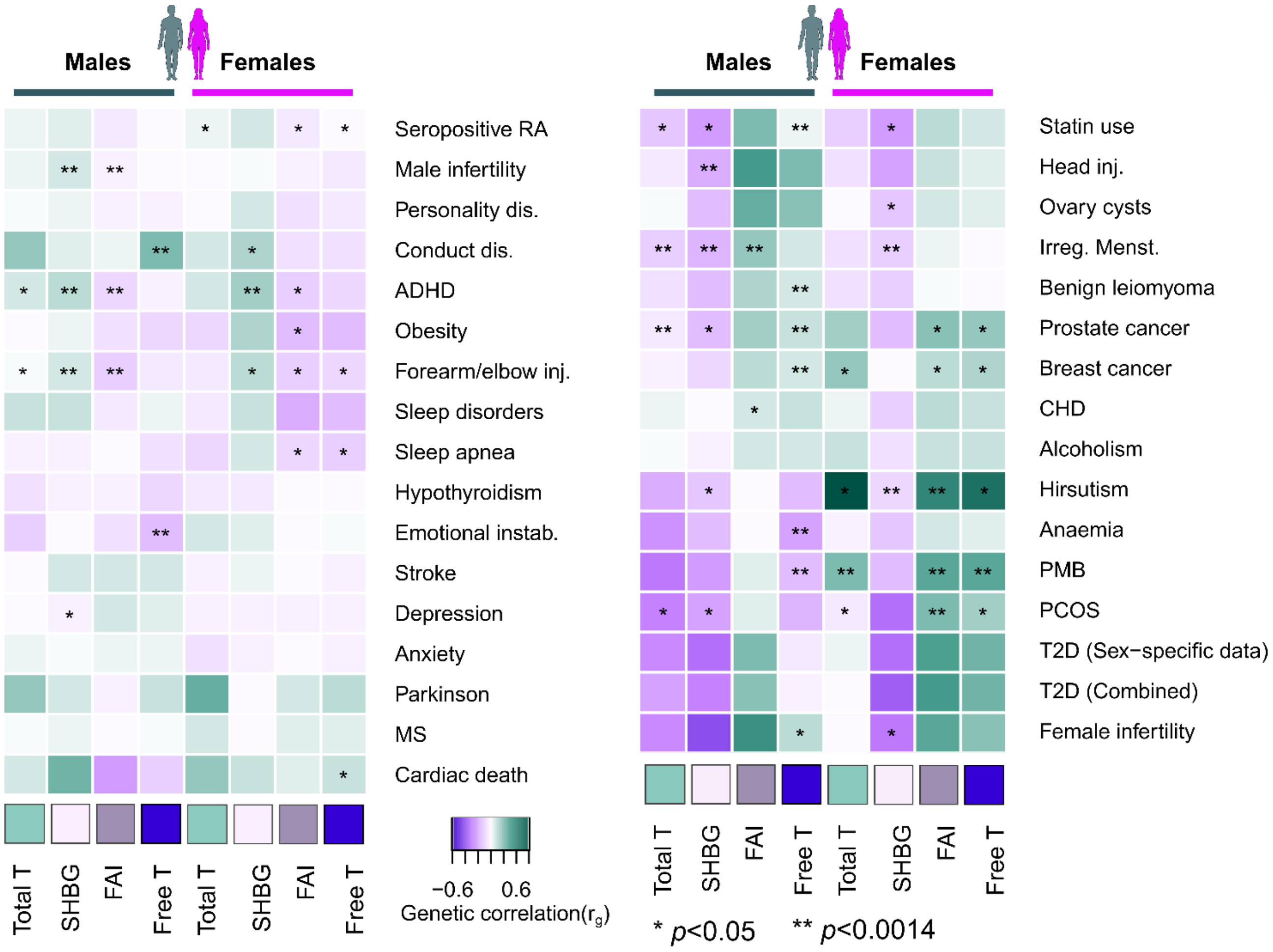

Heatmap colors indicate the direction and the strength of the genetic correlation (purple, negative correlation, green, positive correlation) based on LCV. Green = total testosterone, white lilac = SHBG, grey = free androgen index, blue = free testosterone. Positive genetic correlation means that same genetic variants that increase testosterone/SHBG increase also the risk for endpoint X. Asterisks indicate statistical support (p<0.05 and p<0.0014) for causality in either LCV or MR Egger analyses (Supplementary Data 8). Correlation without causation suggests that the genetic connection between the traits is likely mediated by genetic pleiotropy or that the causal effect is simply too weak to be detected. For both grids, the effects of male PGS on the studied trait is shown on left hand side (under green line), and the effect of the female PGS on the right (under pink line). Please note that this figure also illustrates the effects of male PGS for female traits, and vice versa. The data is based on PGS association analyses comprising of 94,478 men and 122,986 women from FinnGen R5.

Supplementary Figure 10. The effects of body mass index (BMI) and other covariates on testosterone (T) levels and GWAS results in the UK Biobank

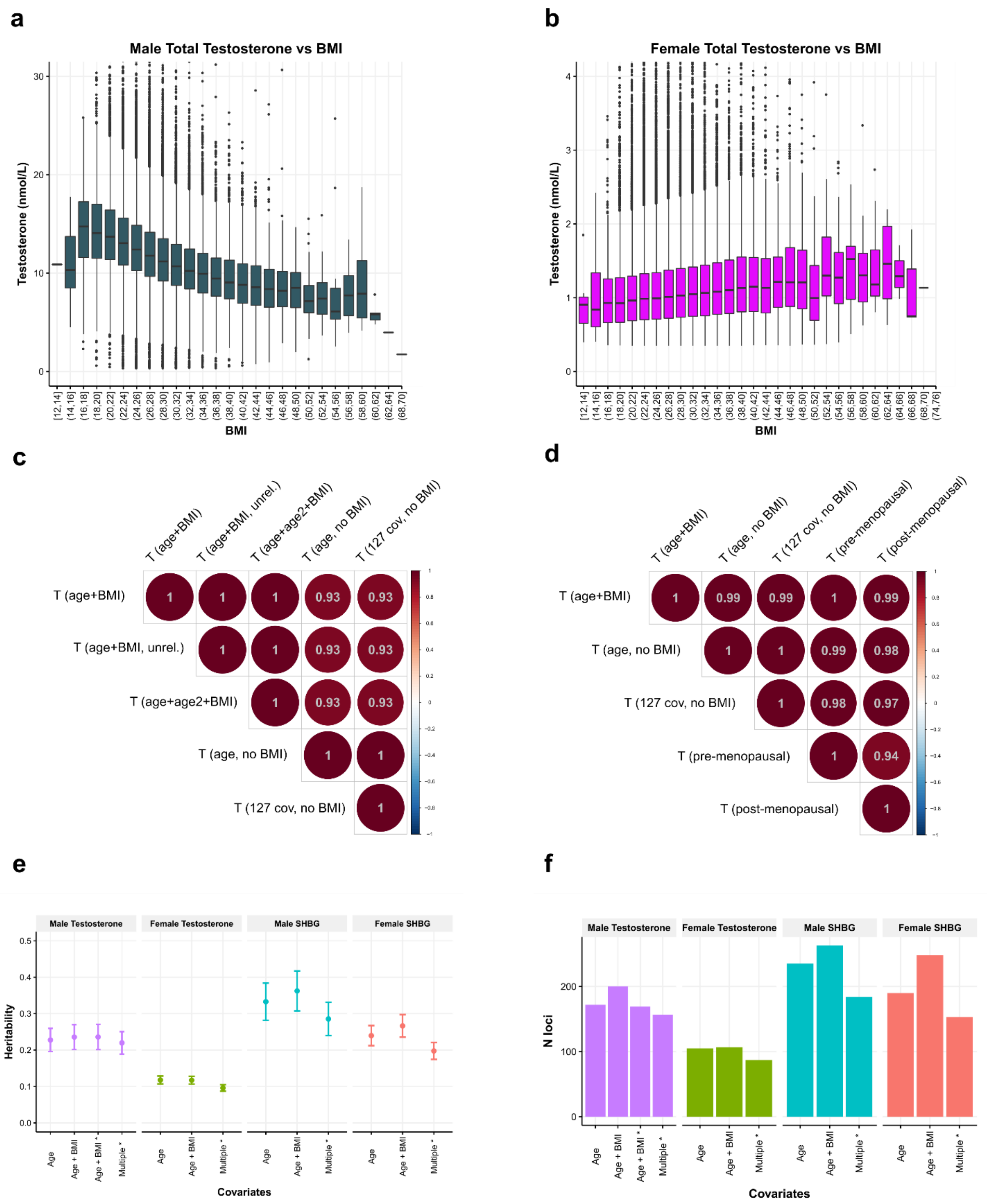

Panels **a** and **b** show the relationship between serum total T levels and BMI (binned in two unit intervals) in the UK Biobank for males (**a,** green boxplots) and females (**b,** pink boxplots), based on 177,186 men and 175,435 women participants with

white British ancestry and testosterone measurements available. For men, a significant negative correlation exists between BMI and T levels (R=-0.295, p=0), whereas this relationship is reversed in women (R=0.081, p=2.9e-230). The box plots show median (black line) and lower and upper quartiles (colored area of the box) for serum total T, and the error bars indicate 5% and 95% quantiles. For visual purposes, the Y-axis is capped at 30nmol/l in men and 4nmol/l in women. Panels **c** and **d** show genetic correlation from LDSC between total T GWAS runs under varying covariate combinations in men (**c,** N=176,212) and women (**d,** N=174,850), implying BMI has the largest effects on results, and that for example menopause status has only negligible effects on the genetic findings. N pre-menopausal women included in study = 48,876; N post-menopausal = 136,236. Dark red indicates high genetic correlation. Adding BMI as a covariate increased both the estimated heritability (LDSC) and the number of loci found for both total T and SHBG. Panel **e** shows heritability estimates with SE**,** and **f** shows the number of significant GWAS hits under different models in a bar plot. Lilac = total testosterone in men, green = total testosterone in women, turquoise = SHBG in men, red = SHBG in women. In panels **e** and **f** * denotes a model with no relatives included in the analysis, and "multiple" refers to including 127 covariates from Sinnott-Armstrong et al. 2019. The results suggest that by using BMI as a covariate we can better capture any genetic variants affecting the studied traits in both sexes, and that the additional 127 covariates (for example, sample dilution factors) have negligible effects on the GWAS results.

Supplementary Information

FinnGen consortium members

Full Name	Affiliation	Role1	Role 2
Aarno Palotie	Institute for Molecular Medicine Finland (FIMM), HiLIFE, University of Helsinki, Helsinki; Broad Institute of MIT and Harvard; Massachusetts General Hospital, Boston MA, United States	Steering Committee	Steering Committee
Mark Daly	Institute for Molecular Medicine Finland (FIMM), HiLIFE, University of Helsinki, Helsinki; Broad Institute of MIT and Harvard; Massachusetts General Hospital, Boston MA, United States	Steering Committee	Steering Committee
Bridget Riley-Gills	Abbvie, Chicago, IL, United States	Steering Committee	Pharmaceutical companies
Howard Jacob	Abbvie, Chicago, IL, United States	Steering Committee	Pharmaceutical companies
Dirk Paul	Astra Zeneca, Cambridge, United Kingdom	Steering Committee	Pharmaceutical companies
Athena Matakidou	Astra Zeneca, Cambridge, United Kingdom	Steering Committee	Pharmaceutical companies
Adam Platt	Astra Zeneca, Cambridge, United Kingdom	Steering Committee	Pharmaceutical companies
Heiko Runz	Biogen, Cambridge, MA, United States	Steering Committee	Pharmaceutical companies
Sally John Biogen,	Cambridge, MA, United States	Steering Committee	Pharmaceutical companies
George Okafo	Boehringer Ingelheim, Ingelheim am Rhein, Germany	Steering Committee	Pharmaceutical companies
Nathan Lawless	Boehringer Ingelheim, Ingelheim am Rhein, Germany	Steering Committee	Pharmaceutical companies
Heli Salminen-Mankonen	Boehringer Ingelheim, Ingelheim am Rhein, German	Steering Committee	Pharmaceutical companies
Robert Plenge	Bristol Myers Squibb, New York, NY, United States	Steering Committee	Pharmaceutical companies
Joseph Maranville	Bristol Myers Squibb, New York, NY, United States	Steering Committee	Pharmaceutical companies
Mark McCarthy	Genentech, San Francisco, CA, United States	Steering Committee	Pharmaceutical companies
Margaret G. Ehm	GlaxoSmithKline, Collegeville, PA, United States	Steering Committee	Pharmaceutical companies
Kirsi Auro	GlaxoSmithKline, Espoo, Finland	Steering Committee	Pharmaceutical companies
Simonne Longerich	Merck, Kenilworth, NJ, United States	Steering Committee	Pharmaceutical companies
Caroline Fox	Merck, Kenilworth, NJ, United States	Steering Committee	Pharmaceutical companies
Anders Mälarstig	Pfizer, New York, NY, United States	Steering Committee	Pharmaceutical companies
Katherine Klinger	Translational Sciences, Sanofi R&D, Framingham, MA, USA	Steering Committee	Pharmaceutical companies
Clement Chatelain	Translational Sciences, Sanofi R&D, Framingham, MA, USA	Steering Committee	Pharmaceutical companies
Matthias Gossel	Translational Sciences, Sanofi R&D, Framingham, MA, USA	Steering Committee	Pharmaceutical companies
Karol Estrada	Maze Therapeutics, San Francisco, CA, United States	Steering Committee	Pharmaceutical companies
Robert Graham	Maze Therapeutics, San Francisco, CA, United States	Steering Committee	Pharmaceutical companies
Robert Yang	Janssen Biotech, Beerse, Belgium	Steering Committee	Pharmaceutical companies
Chris O´Donnell	Novartis Institutes for BioMedical Research, Cambridge, MA, United States	Steering Committee	Pharmaceutical companies
Tomi P. Mäkelä	HiLIFE, University of Helsinki, Finland, Finland	Steering Committee	University of Helsinki & Biobanks
Jaakko Kaprio	Institute for Molecular Medicine Finland (FIMM), HiLIFE, University of Helsinki, Helsinki, Finland	Steering Committee	University of Helsinki & Biobanks
Petri Virolainen	FinlandAuria Biobank / University of Turku / Hospital District of Southwest Finland, Turku,	Steering Committee	University of Helsinki & Biobanks
Antti Hakanen	FinlandAuria Biobank / University of Turku / Hospital District of Southwest Finland, Turku,	Steering Committee	University of Helsinki & Biobanks
Terhi Kilpi	FinlandTHL Biobank / Finnish Institute for Health and Welfare (THL), Helsinki, Finland	Steering Committee	University of Helsinki & Biobanks
Markus Perola	THL Biobank / Finnish Institute for Health and Welfare (THL), Helsinki, Finland	Steering Committee	University of Helsinki & Biobanks
Jukka Partanen	Finnish Red Cross Blood Service / Finnish Hematology Registry and Clinical Biobank, Helsinki, Finland	Steering Committee	University of Helsinki & Biobanks
Anne Pitkäranta	Helsinki Biobank / Helsinki University and Hospital District of Helsinki and Uusimaa, Helsinki, Finland	Steering Committee	University of Helsinki & Biobanks
Taneli Raivio	Helsinki Biobank / Helsinki University and Hospital District of Helsinki and Uusimaa, Helsinki, Finland	Steering Committee	University of Helsinki & Biobanks
Raisa Serpi	Northern Finland Biobank Borealis / University of Oulu / Northern Ostrobothnia Hospital District, Oulu, Finland	Steering Committee	University of Helsinki & Biobanks
Tarja Laitinen	Finnish Clinical Biobank Tampere / University of Tampere / Pirkanmaa Hospital District, Tampere, Finland	Steering Committee	University of Helsinki & Biobanks
Veli-Matti Kosma	Biobank of Eastern Finland / University of Eastern Finland / Northern Savo Hospital District, Kuopio, Finland	Steering Committee	University of Helsinki & Biobanks
Jari Laukkanen	Central Finland Biobank / University of Jyväskylä / Central Finland Health Care District, Jyväskylä, Finland	Steering Committee	University of Helsinki & Biobanks
Marco Hautalahti	FINBB - Finnish biobank cooperative	Steering Committee	University of Helsinki & Biobanks

Outi Tuovila	Business Finland, Helsinki, Finland	Steering Committee	Other Experts/ Non-Voting Members
Raimo Pakkanen	Business Finland, Helsinki, Finland	Steering Committee	Other Experts/ Non-Voting Members
Jeffrey Waring	Abbvie, Chicago, IL, United States	Scientific Committee	Pharmaceutical companies
Bridget Riley-Gillis	Abbvie, Chicago, IL, United States	Scientific Committee	Pharmaceutical companies
Fedik Rahimov	Abbvie, Chicago, IL, United States	Scientific Committee	Pharmaceutical companies
Ioanna Tachmazidou	Astra Zeneca, Cambridge, United Kingdom	Scientific Committee	Pharmaceutical companies
Chia-Yen Chen	Biogen, Cambridge, MA, United States	Scientific Committee	Pharmaceutical companies
Heiko Runz	Biogen, Cambridge, MA, United States	Scientific Committee	Pharmaceutical companies
Zhihao Ding	Boehringer Ingelheim, Ingelheim am Rhein, Germany	Scientific Committee	Pharmaceutical companies
Marc Jung	Boehringer Ingelheim, Ingelheim am Rhein, Germany	Scientific Committee	Pharmaceutical companies
Shameek Biswas	Bristol Myers Squibb, New York, NY, United States	Scientific Committee	Pharmaceutical companies
Rion Pendergrass	Genentech, San Francisco, CA, United States	Scientific Committee	Pharmaceutical companies
Margaret G. Ehm	GlaxoSmithKline, Collegeville, PA, United States	Scientific Committee	Pharmaceutical companies
David Pulford	GlaxoSmithKline, Stevenage, United Kingdom	Scientific Committee	Pharmaceutical companies
Neha Raghavan	Merck, Kenilworth, NJ, United States	Scientific Committee	Pharmaceutical companies
Adriana Huertas-Vazquez	Merck, Kenilworth, NJ, United States	Scientific Committee	Pharmaceutical companies
Jae-Hoon Sul	Merck, Kenilworth, NJ, United States	Scientific Committee	Pharmaceutical companies
Anders Mälarstig	Pfizer, New York, NY, United States	Scientific Committee	Pharmaceutical companies
Xinli Hu	Pfizer, New York, NY, United States	Scientific Committee	Pharmaceutical companies
Åsa Hedman	Pfizer, New York, NY, United States	Scientific Committee	Pharmaceutical companies
Katherine Klinger	Translational Sciences, Sanofi R&D, Framingham, MA, USA	Scientific Committee	Pharmaceutical companies
Robert Graham	Maze Therapeutics, San Francisco, CA, United States	Scientific Committee	Pharmaceutical companies
Manuel Rivas	Maze Therapeutics, San Francisco, CA, United States	Scientific Committee	Pharmaceutical companies
Dawn Waterworth	Janssen Research & Development, LLC, Spring House, PA, United States	Scientific Committee	Pharmaceutical companies
Nicole Renaud	Novartis Institutes for BioMedical Research, Cambridge, MA, United States	Scientific Committee	Pharmaceutical companies
Ma´en Obeidat	Novartis Institutes for BioMedical Research, Cambridge, MA, United States	Scientific Committee	Pharmaceutical companies
Johanna Schleutker	Auria Biobank / Univ. of Turku / Hospital District of Southwest Finland, Turku, Finland	Scientific Committee	University of Helsinki & Biobanks
Markus Perola	THL Biobank / Finnish Institute for Health and Welfare (THL), Helsinki, Finland	Scientific Committee	University of Helsinki & Biobanks
Samuli Ripatti	Institute for Molecular Medicine Finland (FIMM), HiLIFE, University of Helsinki, Helsinki, Finland	Scientific Committee	University of Helsinki & Biobanks
Mikko Arvas	Finnish Red Cross Blood Service / Finnish Hematology Registry and Clinical Biobank, Helsinki, Finland	Scientific Committee	University of Helsinki & Biobanks
Olli Carpén	Helsinki Biobank / Helsinki University and Hospital District of Helsinki and Uusimaa, Helsinki, Finland	Scientific Committee	University of Helsinki & Biobanks
Reetta Hinttala	Northern Finland Biobank Borealis / University of Oulu / Northern Ostrobothnia Hospital District, Oulu, Finland	Scientific Committee	University of Helsinki & Biobanks
Johannes Kettunen	Northern Finland Biobank Borealis / University of Oulu / Northern Ostrobothnia Hospital District, Oulu, Finland	Scientific Committee	University of Helsinki & Biobanks
Arto Mannermaa	Biobank of Eastern Finland / University of Eastern Finland / Northern Savo Hospital District, Kuopio, Finland	Scientific Committee	University of Helsinki & Biobanks
Katriina Aalto-Setälä	Faculty of Medicine and Health Technology, Tampere University, Tampere, Finland	Scientific Committee	University of Helsinki & Biobanks
Mika Kähönen	Finnish Clinical Biobank Tampere / University of Tampere / Pirkanmaa Hospital District, Tampere, Finland	Scientific Committee	University of Helsinki & Biobanks
Jari Laukkanen	Central Finland Biobank / University of Jyväskylä / Central Finland Health Care District, Jyväskylä, Finland	Scientific Committee	University of Helsinki & Biobanks
Johanna Mäkelä	FINBB - Finnish biobank cooperative	Scientific Committee	University of Helsinki & Biobanks
Reetta Kälviäinen	Northern Savo Hospital District, Kuopio, Finland	Clinical Groups	Neurology Group
Valtteri Julkunen	Northern Savo Hospital District, Kuopio, Finland	Clinical Groups	Neurology Group
Hilkka Soininen	Northern Savo Hospital District, Kuopio, Finland	Clinical Groups	Neurology Group
Anne Remes	Northern Ostrobothnia Hospital District, Oulu, Finland	Clinical Groups	Neurology Group
Mikko Hiltunen	University of Eastern Finland, Kuopio, Finland	Clinical Groups	Neurology Group
Jukka Peltola	Pirkanmaa Hospital District, Tampere, Finland	Clinical Groups	Neurology Group

Minna Raivio	Hospital District of Helsinki and Uusimaa, Helsinki, Finland	Clinical Groups	Neurology Group
Pentti Tienari	Hospital District of Helsinki and Uusimaa, Helsinki, Finland	Clinical Groups	Neurology Group
Juha Rinne	Hospital District of Southwest Finland, Turku, Finland	Clinical Groups	Neurology Group
Roosa Kallionpää	Hospital District of Southwest Finland, Turku, Finland	Clinical Groups	Neurology Group
Juulia Partanen	Institute for Molecular Medicine Finland, HiLIFE, University of Helsinki, Finland	Clinical Groups	Neurology Group
Ali Abbasi	Abbvie, Chicago, IL, United States	Clinical Groups	Neurology Group
Adam Ziemann	Abbvie, Chicago, IL, United States	Clinical Groups	Neurology Group
Nizar Smaoui	Abbvie, Chicago, IL, United States	Clinical Groups	Neurology Group
Anne Lehtonen	Abbvie, Chicago, IL, United States	Clinical Groups	Neurology Group
Susan Eaton	Biogen, Cambridge, MA, United States	Clinical Groups	Neurology Group
Heiko Runz	Biogen, Cambridge, MA, United States	Clinical Groups	Neurology Group
Sanni Lahdenperä	Biogen, Cambridge, MA, United States	Clinical Groups	Neurology Group
Shameek Biswas	Bristol Myers Squibb, New York, NY, United States	Clinical Groups	Neurology Group
Natalie Bowers	Genentech, San Francisco, CA, United States	Clinical Groups	Neurology Group
Edmond Teng	Genentech, San Francisco, CA, United States	Clinical Groups	Neurology Group
Rion Pendergrass	Genentech, San Francisco, CA, United States	Clinical Groups	Neurology Group
Fanli Xu	GlaxoSmithKline, Brentford, United Kingdom	Clinical Groups	Neurology Group
David Pulford	GlaxoSmithKline, Stevenage, United Kingdom	Clinical Groups	Neurology Group
Kirsi Auro	GlaxoSmithKline, Espoo, Finland	Clinical Groups	Neurology Group
Laura Addis	GlaxoSmithKline, Brentford, United Kingdom	Clinical Groups	Neurology Group
John Eicher	GlaxoSmithKline, Brentford, United Kingdom	Clinical Groups	Neurology Group
Qingqin S Li	Janssen Research & Development, LLC, Titusville, NJ 08560, United States	Clinical Groups	Neurology Group
Karen He	Janssen Research & Development, LLC, Spring House, PA, United States	Clinical Groups	Neurology Group
Ekaterina Khramtsova	Janssen Research & Development, LLC, Spring House, PA, United States	Clinical Groups	Neurology Group
Neha Raghavan	Merck, Kenilworth, NJ, United States	Clinical Groups	Neurology Group
Martti Färkkilä	Hospital District of Helsinki and Uusimaa, Helsinki, Finland	Clinical Groups	Gastroenterology Group
Jukka Koskela	Hospital District of Helsinki and Uusimaa, Helsinki, Finland	Clinical Groups	Gastroenterology Group
Sampsa Pikkarainen	Hospital District of Helsinki and Uusimaa, Helsinki, Finland	Clinical Groups	Gastroenterology Group
Airi Jussila	Pirkanmaa Hospital District, Tampere, Finland	Clinical Groups	Gastroenterology Group
Katri Kaukinen	Pirkanmaa Hospital District, Tampere, Finland	Clinical Groups	Gastroenterology Group
Timo Blomster	Northern Ostrobothnia Hospital District, Oulu, Finland	Clinical Groups	Gastroenterology Group
Mikko Kiviniemi	Northern Savo Hospital District, Kuopio, Finland	Clinical Groups	Gastroenterology Group
Markku Voutilainen	Hospital District of Southwest Finland, Turku, Finland	Clinical Groups	Gastroenterology Group
Mark Daly	Institute for Molecular Medicine Finland (FIMM), HiLIFE, University of Helsinki, Helsinki; Broad Institute of MIT and Harvard; Massachusetts General Hospital, Boston MA, United States	Clinical Groups	Gastroenterology Group
Ali Abbasi	Abbvie, Chicago, IL, United States	Clinical Groups	Gastroenterology Group
Jeffrey Waring	Abbvie, Chicago, IL, United States	Clinical Groups	Gastroenterology Group
Nizar Smaoui	Abbvie, Chicago, IL, United States	Clinical Groups	Gastroenterology Group
Fedik Rahimov	Abbvie, Chicago, IL, United States	Clinical Groups	Gastroenterology Group
Anne Lehtonen	Abbvie, Chicago, IL, United States	Clinical Groups	Gastroenterology Group
Tim Lu	Genentech, San Francisco, CA, United States	Clinical Groups	Gastroenterology Group
Natalie Bowers	Genentech, San Francisco, CA, United States	Clinical Groups	Gastroenterology Group
Rion Pendergrass	Genentech, San Francisco, CA, United States	Clinical Groups	Gastroenterology Group
Linda McCarthy	GlaxoSmithKline, Brentford, United Kingdom	Clinical Groups	Gastroenterology Group
Amy Hart	Janssen Research & Development, LLC, Spring House, PA, United States	Clinical Groups	Gastroenterology Group

Meijian Guan	Janssen Research & Development, LLC, Spring House, PA, United States	Clinical Groups	Gastroenterology Group
Jason Miller	Merck, Kenilworth, NJ, United States	Clinical Groups	Gastroenterology Group
Kirsi Kalpala	Pfizer, New York, NY, United States	Clinical Groups	Gastroenterology Group
Melissa Miller	Pfizer, New York, NY, United States	Clinical Groups	Gastroenterology Group
Xinli Hu	Pfizer, New York, NY, United States	Clinical Groups	Gastroenterology Group
Kari Eklund	Hospital District of Helsinki and Uusimaa, Helsinki, Finland	Clinical Groups	Rheumatology Group
Antti Palomäki	Hospital District of Southwest Finland, Turku, Finland	Clinical Groups	Rheumatology Group
Pia Isomäki	Pirkanmaa Hospital District, Tampere, Finland	Clinical Groups	Rheumatology Group
Laura Pirilä	Hospital District of Southwest Finland, Turku, Finland	Clinical Groups	Rheumatology Group
Oili Kaipiainen-Seppänen	Northern Savo Hospital District, Kuopio, Finland	Clinical Groups	Rheumatology Group
Johanna Huhtakangas	Northern Ostrobothnia Hospital District, Oulu, Finland	Clinical Groups	Rheumatology Group
Nina Mars	Institute for Molecular Medicine Finland (FIMM), HiLIFE, University of Helsinki, Helsinki, Finland	Clinical Groups	Rheumatology Group
Ali Abbasi	Abbvie, Chicago, IL, United States	Clinical Groups	Rheumatology Group
Jeffrey Waring	Abbvie, Chicago, IL, United States	Clinical Groups	Rheumatology Group
Fedik Rahimov	Abbvie, Chicago, IL, United States	Clinical Groups	Rheumatology Group
Apinya Lertratanakul	Abbvie, Chicago, IL, United States	Clinical Groups	Rheumatology Group
Nizar Smaoui	Abbvie, Chicago, IL, United States	Clinical Groups	Rheumatology Group
Anne Lehtonen	Abbvie, Chicago, IL, United States	Clinical Groups	Rheumatology Group
Marla Hochfeld	Bristol Myers Squibb, New York, NY, United States	Clinical Groups	Rheumatology Group
Natalie Bowers	Genentech, San Francisco, CA, United States	Clinical Groups	Rheumatology Group
Rion Pendergrass	Genentech, San Francisco, CA, United States	Clinical Groups	Rheumatology Group
Jorge Esparza Gordillo	GlaxoSmithKline, Brentford, United Kingdom	Clinical Groups	Rheumatology Group
Kirsi Auro	GlaxoSmithKline, Espoo, Finland	Clinical Groups	Rheumatology Group
Dawn Waterworth	Janssen Research & Development, LLC, Spring House, PA, United States	Clinical Groups	Rheumatology Group
Fabiana Farias	Merck, Kenilworth, NJ, United States	Clinical Groups	Rheumatology Group
Kirsi Kalpala	Pfizer, New York, NY, United States	Clinical Groups	Rheumatology Group
Nan Bing	Pfizer, New York, NY, United States	Clinical Groups	Rheumatology Group
Xinli Hu	Pfizer, New York, NY, United States	Clinical Groups	Rheumatology Group
Tarja Laitinen	Pirkanmaa Hospital District, Tampere, Finland	Clinical Groups	Pulmonology Group
Margit Pelkonen	Northern Savo Hospital District, Kuopio, Finland	Clinical Groups	Pulmonology Group
Paula Kauppi	Hospital District of Helsinki and Uusimaa, Helsinki, Finland	Clinical Groups	Pulmonology Group
Hannu Kankaanranta	University of Gothenburg, Gothenburg, Sweden/ Seinäjoki Central Hospital, Seinäjoki, Finland/ Tampere University, Tampere, Finland	Clinical Groups	Pulmonology Group
Terttu Harju	Northern Ostrobothnia Hospital District, Oulu, Finland	Clinical Groups	Pulmonology Group
Riitta Lahesmaa	Hospital District of Southwest Finland, Turku, Finland	Clinical Groups	Pulmonology Group
Nizar Smaoui	Abbvie, Chicago, IL, United States	Clinical Groups	Pulmonology Group
Glenda Lassi	Astra Zeneca, Cambridge, United Kingdom	Clinical Groups	Pulmonology Group
Susan Eaton	Biogen, Cambridge, MA, United States	Clinical Groups	Pulmonology Group
Hubert Chen	Genentech, San Francisco, CA, United States	Clinical Groups	Pulmonology Group
Rion Pendergrass	Genentech, San Francisco, CA, United States	Clinical Groups	Pulmonology Group
Natalie Bowers	Genentech, San Francisco, CA, United States	Clinical Groups	Pulmonology Group
Joanna Betts	GlaxoSmithKline, Brentford, United Kingdom	Clinical Groups	Pulmonology Group
Kirsi Auro	GlaxoSmithKline, Espoo, Finland	Clinical Groups	Pulmonology Group
Rajashree Mishra	GlaxoSmithKline, Brentford, United Kingdom	Clinical Groups	Pulmonology Group
Majd Mouded	Novartis, Basel, Switzerland	Clinical Groups	Pulmonology Group

Debby Ngo	Novartis, Basel, Switzerland	Clinical Groups	Pulmonology Group
Teemu Niiranen	Finnish Institute for Health and Welfare (THL), Helsinki, Finland	Clinical Groups	Cardiometabolic Diseases Group
Felix Vaura	Finnish Institute for Health and Welfare (THL), Helsinki, Finland	Clinical Groups	Cardiometabolic Diseases Group
Veikko Salomaa	Finnish Institute for Health and Welfare (THL), Helsinki, Finland	Clinical Groups	Cardiometabolic Diseases Group
Kaj Metsärinne	Hospital District of Southwest Finland, Turku, Finland	Clinical Groups	Cardiometabolic Diseases Group
Jenni Aittokallio	Hospital District of Southwest Finland, Turku, Finland	Clinical Groups	Cardiometabolic Diseases Group
Jussi Hernesniemi	Pirkanmaa Hospital District, Tampere, Finland	Clinical Groups	Cardiometabolic Diseases Group
Daniel Gordin	Hospital District of Helsinki and Uusimaa, Helsinki, Finland	Clinical Groups	Cardiometabolic Diseases Group
Juha Sinisalo	Hospital District of Helsinki and Uusimaa, Helsinki, Finland	Clinical Groups	Cardiometabolic Diseases Group
Marja-Riitta Taskinen	Hospital District of Helsinki and Uusimaa, Helsinki, Finland	Clinical Groups	Cardiometabolic Diseases Group
Tiinamaija Tuomi	Hospital District of Helsinki and Uusimaa, Helsinki, Finland	Clinical Groups	Cardiometabolic Diseases Group
Timo Hiltunen	Hospital District of Helsinki and Uusimaa, Helsinki, Finland	Clinical Groups	Cardiometabolic Diseases Group
Jari Laukkanen	Central Finland Health Care District, Jyväskylä, Finland	Clinical Groups	Cardiometabolic Diseases Group
Amanda Elliott	Institute for Molecular Medicine Finland (FIMM), HiLIFE, University of Helsinki, Helsinki, Finland; Broad Institute, Cambridge, MA, USA and Massachusetts General Hospital, Boston, MA, USA	Clinical Groups	Cardiometabolic Diseases Group
Mary Pat Reeve	Institute for Molecular Medicine Finland (FIMM), HiLIFE, University of Helsinki, Helsinki, Finland	Clinical Groups	Cardiometabolic Diseases Group
Sanni Ruotsalainen	Institute for Molecular Medicine Finland (FIMM), HiLIFE, University of Helsinki, Helsinki, Finland	Clinical Groups	Cardiometabolic Diseases Group
Benjamin Challis	Astra Zeneca, Cambridge, United Kingdom	Clinical Groups	Cardiometabolic Diseases Group
Dirk Paul	Astra Zeneca, Cambridge, United Kingdom	Clinical Groups	Cardiometabolic Diseases Group
Natalie Bowers	Genentech, San Francisco, CA, United States	Clinical Groups	Cardiometabolic Diseases Group
Rion Pendergrass	Genentech, San Francisco, CA, United States	Clinical Groups	Cardiometabolic Diseases Group
Audrey Chu	GlaxoSmithKline, Brentford, United Kingdom	Clinical Groups	Cardiometabolic Diseases Group
Kirsi Auro	GlaxoSmithKline, Espoo, Finland	Clinical Groups	Cardiometabolic Diseases Group
Dermot Reilly	Janssen Research & Development, LLC, Boston, MA, United States	Clinical Groups	Cardiometabolic Diseases Group
Mike Mendelson	Novartis, Boston, MA, United States	Clinical Groups	Cardiometabolic Diseases Group
Jaakko Parkkinen	Pfizer, New York, NY, United States	Clinical Groups	Cardiometabolic Diseases Group
Melissa Miller	Pfizer, New York, NY, United States	Clinical Groups	Cardiometabolic Diseases Group
Tuomo Meretoja	Hospital District of Helsinki and Uusimaa, Helsinki, Finland	Clinical Groups	Oncology Group
Heikki Joensuu	Hospital District of Helsinki and Uusimaa, Helsinki, Finland	Clinical Groups	Oncology Group
Olli Carpén	Hospital District of Helsinki and Uusimaa, Helsinki, Finland	Clinical Groups	Oncology Group
Johanna Mattson	Hospital District of Helsinki and Uusimaa, Helsinki, Finland	Clinical Groups	Oncology Group
Eveliina Salminen	Hospital District of Helsinki and Uusimaa, Helsinki, Finland	Clinical Groups	Oncology Group
Annika Auranen	Pirkanmaa Hospital District , Tampere, Finland	Clinical Groups	Oncology Group
Peeter Karihtala	Northern Ostrobothnia Hospital District, Oulu, Finland	Clinical Groups	Oncology Group
Päivi Auvinen	Northern Savo Hospital District, Kuopio, Finland	Clinical Groups	Oncology Group
Klaus Elenius	Hospital District of Southwest Finland, Turku, Finland	Clinical Groups	Oncology Group
Johanna Schleutker	Hospital District of Southwest Finland, Turku, Finland	Clinical Groups	Oncology Group
Esa Pitkänen	Institute for Molecular Medicine Finland (FIMM), HiLIFE, University of Helsinki, Helsinki, Finland	Clinical Groups	Oncology Group
Mark Daly	Institute for Molecular Medicine Finland (FIMM), HiLIFE, University of Helsinki, Helsinki; Broad Institute of MIT and Harvard; Massachusetts General Hospital, Boston MA, United States	Clinical Groups	Oncology Group
Nina Mars	Institute for Molecular Medicine Finland (FIMM), HiLIFE, University of Helsinki, Helsinki, Finland	Clinical Groups	Oncology Group
Relja Popovic	Abbvie, Chicago, IL, United States	Clinical Groups	Oncology Group
Jeffrey Waring	Abbvie, Chicago, IL, United States	Clinical Groups	Oncology Group
Bridget Riley-Gillis	Abbvie, Chicago, IL, United States	Clinical Groups	Oncology Group
Anne Lehtonen	Abbvie, Chicago, IL, United States	Clinical Groups	Oncology Group
Jennifer Schutzman	Genentech, San Francisco, CA, United States	Clinical Groups	Oncology Group

Natalie Bowers	Genentech, San Francisco, CA, United States	Clinical Groups	Oncology Group
Rion Pendergrass	Genentech, San Francisco, CA, United States	Clinical Groups	Oncology Group
Diptee Kulkarni	GlaxoSmithKline, Brentford, United Kingdom	Clinical Groups	Oncology Group
Kirsi Auro	GlaxoSmithKline, Espoo, Finland	Clinical Groups	Oncology Group
Alessandro Porello	Janssen Research & Development, LLC, Spring House, PA, United States	Clinical Groups	Oncology Group
Andrey Loboda	Merck, Kenilworth, NJ, United States	Clinical Groups	Oncology Group
Heli Lehtonen	Pfizer, New York, NY, United States	Clinical Groups	Oncology Group
Stefan McDonough	Pfizer, New York, NY, United States	Clinical Groups	Oncology Group
Sauli Vuoti	Janssen-Cilag Oy, Espoo, Finland	Clinical Groups	Oncology Group
Kai Kaarniranta	Northern Savo Hospital District, Kuopio, Finland	Clinical Groups	Opthalmology Group
Joni A Turunen	Helsinki University Hospital and University of Helsinki, Helsinki, Finland; Eye Genetics Group, Folkhälsan Research Center, Helsinki, Finland	Clinical Groups	Opthalmology Group
Terhi Ollila	Hospital District of Helsinki and Uusimaa, Helsinki, Finland	Clinical Groups	Opthalmology Group
Hannu Uusitalo	Pirkanmaa Hospital District, Tampere, Finland	Clinical Groups	Opthalmology Group
Esa Pitkänen	Institute for Molecular Medicine Finland (FIMM), HiLIFE, University of Helsinki, Helsinki, Finland	Clinical Groups	Opthalmology Group
Juha Karjalainen	Institute for Molecular Medicine Finland (FIMM), HiLIFE, University of Helsinki, Helsinki, Finland	Clinical Groups	Opthalmology Group
Mengzhen Liu	Abbvie, Chicago, IL, United States	Clinical Groups	Opthalmology Group
Heiko Runz	Biogen, Cambridge, MA, United States	Clinical Groups	Opthalmology Group
Stephanie Loomis s	Biogen, Cambridge, MA, United State	Clinical Groups	Opthalmology Group
Erich Strauss	Genentech, San Francisco, CA, United States	Clinical Groups	Opthalmology Group
Natalie Bowers	Genentech, San Francisco, CA, United States	Clinical Groups	Opthalmology Group
Hao Chen	Genentech, San Francisco, CA, United States	Clinical Groups	Opthalmology Group
Rion Pendergrass	Genentech, San Francisco, CA, United States	Clinical Groups	Opthalmology Group
Kaisa Tasanen	Northern Ostrobothnia Hospital District, Oulu, Finland	Clinical Groups	Dermatology Group
Laura Huilaja	Northern Ostrobothnia Hospital District, Oulu, Finland	Clinical Groups	Dermatology Group
Katariina Hannula-Jouppi	Hospital District of Helsinki and Uusimaa, Helsinki, Finland	Clinical Groups	Dermatology Group
Teea Salmi	Pirkanmaa Hospital District, Tampere, Finland	Clinical Groups	Dermatology Group
Sirkku Peltonen	Hospital District of Southwest Finland, Turku, Finland	Clinical Groups	Dermatology Group
Leena Koulu	Hospital District of Southwest Finland, Turku, Finland	Clinical Groups	Dermatology Group
Nizar Smaoui	Abbvie, Chicago, IL, United States	Clinical Groups	Dermatology Group
Fedik Rahimov	Abbvie, Chicago, IL, United States	Clinical Groups	Dermatology Group
Anne Lehtonen	Abbvie, Chicago, IL, United States	Clinical Groups	Dermatology Group
David Choy	Genentech, San Francisco, CA, United States	Clinical Groups	Dermatology Group
Rion Pendergrass	Genentech, San Francisco, CA, United States	Clinical Groups	Dermatology Group
Dawn Waterworth	Janssen Research & Development, LLC, Spring House, PA, United States	Clinical Groups	Dermatology Group
Kirsi Kalpala	Pfizer, New York, NY, United States	Clinical Groups	Dermatology Group
Ying Wu	Pfizer, New York, NY, United States	Clinical Groups	Dermatology Group
Pirkko Pussinen	Hospital District of Helsinki and Uusimaa, Helsinki, Finland	Clinical Groups	Odontology Group
Aino Salminen	Hospital District of Helsinki and Uusimaa, Helsinki, Finland	Clinical Groups	Odontology Group
Tuula Salo	Hospital District of Helsinki and Uusimaa, Helsinki, Finland	Clinical Groups	Odontology Group
David Rice	Hospital District of Helsinki and Uusimaa, Helsinki, Finland	Clinical Groups	Odontology Group
Pekka Nieminen	Hospital District of Helsinki and Uusimaa, Helsinki, Finland	Clinical Groups	Odontology Group
Ulla Palotie	Hospital District of Helsinki and Uusimaa, Helsinki, Finland	Clinical Groups	Odontology Group
Maria Siponen	Northern Savo Hospital District, Kuopio, Finland	Clinical Groups	Odontology Group
Liisa Suominen	Northern Savo Hospital District, Kuopio, Finland	Clinical Groups	Odontology Group

Päivi Mäntylä	Northern Savo Hospital District, Kuopio, Finland	Clinical Groups	Odontology Group
Ulvi Gursoy	Hospital District of Southwest Finland, Turku, Finland	Clinical Groups	Odontology Group
Vuokko Anttonen	Northern Ostrobothnia Hospital District, Oulu, Finland	Clinical Groups	Odontology Group
Kirsi Sipilä	Research Unit of Oral Health Sciences Faculty of Medicine, University of Oulu, Oulu, Finland; Medical Research Center, Oulu, Oulu University Hospital and University of Oulu, Oulu, Finland	Clinical Groups	Odontology Group
Rion Pendergrass	Genentech, San Francisco, CA, United States	Clinical Groups	Odontology Group
Hannele Laivuori	Institute for Molecular Medicine Finland (FIMM), HiLIFE, University of Helsinki, Helsinki, Finland	Clinical Groups	Women's Health and Reproduction Group
Venla Kurra	Pirkanmaa Hospital District, Tampere, Finland	Clinical Groups	Women's Health and Reproduction Group
Laura Kotaniemi-Talonen	Pirkanmaa Hospital District, Tampere, Finland	Clinical Groups	Women's Health and Reproduction Group
Oskari Heikinheimo	Hospital District of Helsinki and Uusimaa, Helsinki, Finland	Clinical Groups	Women's Health and Reproduction Group
Ilkka Kalliala	Hospital District of Helsinki and Uusimaa, Helsinki, Finland	Clinical Groups	Women's Health and Reproduction Group
Lauri Aaltonen	Hospital District of Helsinki and Uusimaa, Helsinki, Finland	Clinical Groups	Women's Health and Reproduction Group
Varpu Jokimaa	Hospital District of Southwest Finland, Turku, Finland	Clinical Groups	Women's Health and Reproduction Group
Terhi Piltonen	Northern Ostrobothnia Hospital District, Oulu, Finland	Clinical Groups	Women's Health and Reproduction Group
Johannes Kettunen	Northern Ostrobothnia Hospital District, Oulu, Finland	Clinical Groups	Women's Health and Reproduction Group
Marja Vääräsmäki	Northern Ostrobothnia Hospital District, Oulu, Finland	Clinical Groups	Women's Health and Reproduction Group
Outi Uimari	Northern Ostrobothnia Hospital District, Oulu, Finland	Clinical Groups	Women's Health and Reproduction Group
Laure Morin-Papunen	Northern Ostrobothnia Hospital District, Oulu, Finland	Clinical Groups	Women's Health and Reproduction Group
Maarit Niinimäki	Northern Ostrobothnia Hospital District, Oulu, Finland	Clinical Groups	Women's Health and Reproduction Group
Katja Kivinen	Institute for Molecular Medicine Finland (FIMM), HiLIFE, University of Helsinki, Helsinki, Finland	Clinical Groups	Women's Health and Reproduction Group
Elisabeth Widen	Institute for Molecular Medicine Finland (FIMM), HiLIFE, University of Helsinki, Helsinki, Finland	Clinical Groups	Women's Health and Reproduction Group
Mary Pat Reeve	Institute for Molecular Medicine Finland (FIMM), HiLIFE, University of Helsinki, Helsinki, Finland	Clinical Groups	Women's Health and Reproduction Group
Mark Daly	Institute for Molecular Medicine Finland (FIMM), HiLIFE, University of Helsinki, Helsinki; Broad Institute of MIT and Harvard; Massachusetts General Hospital, Boston MA, United States	Clinical Groups	Women's Health and Reproduction Group
Taru Tukiainen	Institute for Molecular Medicine Finland (FIMM), HiLIFE, University of Helsinki, Helsinki, Finland	Clinical Groups	Women's Health and Reproduction Group
Niko Välimäki	University of Helsinki, Helsinki, Finland	Clinical Groups	Women's Health and Reproduction Group
Eija Laakkonen	University of Jyväskylä, Jyväskylä, Finland	Clinical Groups	Women's Health and Reproduction Group
Jaakko Tyrmi	University of Oulu, Oulu, Finland / University of Tampere, Tampere, Finland	Clinical Groups	Women's Health and Reproduction Group
Heidi Silven	University of Oulu, Oulu, Finland	Clinical Groups	Women's Health and Reproduction Group
Eeva Sliz	University of Oulu, Oulu, Finland	Clinical Groups	Women's Health and Reproduction Group
Riikka Arffman	University of Oulu, Oulu, Finland	Clinical Groups	Women's Health and Reproduction Group
Susanna Savukoski	University of Oulu, Oulu, Finland	Clinical Groups	Women's Health and Reproduction Group
Triin Laisk	Estonian biobank, Tartu, Estonia	Clinical Groups	Women's Health and Reproduction Group
Natalia Pujol	Estonian biobank, Tartu, Estonia	Clinical Groups	Women's Health and Reproduction Group
Mengzhen Liu	Abbvie, Chicago, IL, United States	Clinical Groups	Women's Health and Reproduction Group
Bridget Riley-Gillis	Abbvie, Chicago, IL, United States	Clinical Groups	Women's Health and Reproduction Group
Rion Pendergrass	Genentech, San Francisco, CA, United States	Clinical Groups	Women's Health and Reproduction Group
Janet Kumar	GlaxoSmithKline, Collegeville, PA, United States	Clinical Groups	Women's Health and Reproduction Group
Kirsi Auro	GlaxoSmithKline, Espoo, Finland	Clinical Groups	Women's Health and Reproduction Group
Iiris Hovatta	University of Helsinki, Finland	Clinical Groups	Depression group
Chia-Yen Chen	Biogen, Cambridge, MA, United States	Clinical Groups	Depression group
Erkki Isometsä	Hospital District of Helsinki and Uusimaa, Helsinki, Finland	Clinical Groups	Depression group
Hanna Ollila	Institute for Molecular Medicine Finland (FIMM), HiLIFE, University of Helsinki, Helsinki, Finland	Clinical Groups	Depression group
Jaana Suvisaari	Finnish Institute for Health and Welfare (THL), Helsinki, Finland	Clinical Groups	Depression group
Thomas Damm Als	Aarhus University, Denmark	Clinical Groups	Depression group
Antti Mäkitie	Department of Otorhinolaryngology - Head and Neck Surgery, University of Helsinki and Helsinki University Hospital, Helsinki, Finland	Clinical Groups	ENT (ear, nose and throath) Group

Argyro Bizaki-Vallaskanga	Pirkanmaa Hospital District, Tampere, Finland	Clinical Groups	ENT (ear, nose and throath) Group
Sanna Toppila-Salmi	University of Helsinki, Finland	Clinical Groups	ENT (ear, nose and throath) Group
Elmo Saarentaus	Institute for Molecular Medicine Finland (FIMM), HiLIFE, University of Helsinki, Helsinki, Finland	Clinical Groups	ENT (ear, nose and throath) Group
Tytti Willberg	Hospital District of Southwest Finland, Turku, Finland	Clinical Groups	ENT (ear, nose and throath) Group
Antti Aarnisalo	Hospital District of Helsinki and Uusimaa, Helsinki, Finland	Clinical Groups	ENT (ear, nose and throath) Group
Eveliina Salminen	Hospital District of Helsinki and Uusimaa, Helsinki, Finland	Clinical Groups	ENT (ear, nose and throath) Group
Elisa Rahikkala	Northern Ostrobothnia Hospital District, Oulu, Finland	Clinical Groups	ENT (ear, nose and throath) Group
Johannes Kettunen	Northern Ostrobothnia Hospital District, Oulu, Finland	Clinical Groups	ENT (ear, nose and throath) Group
Kristiina Aittomäki	Department of Medical Genetics, Helsinki University Central Hospital, Helsinki, Finland	Clinical Groups	POI (premature ovarian failure) Group
Fredrik Åberg	Transplantation and Liver Surgery Clinic, Helsinki University Hospital, Helsinki University, Helsinki, Finland	Clinical Groups	LiverScore Group
Aarno Palotie	Institute for Molecular Medicine, Finland (FIMM), HiLIFE, University of Helsinki, Helsinki, Finland; Broad Institute of MIT and Harvard; Massachusetts General Hospital	FinnGen Analysis working group	FinnGen Analysis working group
Mark Daly	Institute for Molecular Medicine Finland (FIMM), HiLIFE, University of Helsinki, Helsinki; Broad Institute of MIT and Harvard; Massachusetts General Hospital, Boston MA, United States	FinnGen Analysis working group	FinnGen Analysis working group
Samuli Ripatti	Institute for Molecular Medicine Finland (FIMM), HiLIFE, University of Helsinki, Helsinki, Finland	FinnGen Analysis working group	FinnGen Analysis working group
Aki Havulinna	Institute for Molecular Medicine Finland (FIMM), HiLIFE, University of Helsinki, Helsinki, Finland	FinnGen Analysis working group	FinnGen Analysis working group
Mitja Kurki	Institute for Molecular Medicine Finland (FIMM), HiLIFE, University of Helsinki, Helsinki, Finland	FinnGen Analysis working group	FinnGen Analysis working group
Juha Mehtonen	Institute for Molecular Medicine Finland (FIMM), HiLIFE, University of Helsinki, Helsinki, Finland	FinnGen Analysis working group	FinnGen Analysis working group
Priit Palta	Institute for Molecular Medicine Finland (FIMM), HiLIFE, University of Helsinki, Helsinki, Finland	FinnGen Analysis working group	FinnGen Analysis working group
Juha Karjalainen	Institute for Molecular Medicine Finland (FIMM), HiLIFE, University of Helsinki, Helsinki, Finland	FinnGen Analysis working group	FinnGen Analysis working group
Pietro Della Briotta Parolo	Institute for Molecular Medicine Finland (FIMM), HiLIFE, University of Helsinki, Helsinki, Finland	FinnGen Analysis working group	FinnGen Analysis working group
Wei Zhou	Broad Institute, Cambridge, MA, United States	FinnGen Analysis working group	FinnGen Analysis working group
Mutaamba Maasha	Broad Institute, Cambridge, MA, United States	FinnGen Analysis working group	FinnGen Analysis working group
Susanna Lemmelä	Institute for Molecular Medicine Finland (FIMM), HiLIFE, University of Helsinki, Helsinki, Finland	FinnGen Analysis working group	FinnGen Analysis working group
Manuel Rivas	University of Stanford, Stanford, CA, United States	FinnGen Analysis working group	FinnGen Analysis working group
Aoxing Liu	Institute for Molecular Medicine Finland (FIMM), HiLIFE, University of Helsinki, Helsinki, Finland	FinnGen Analysis working group	FinnGen Analysis working group
Arto Lehisto	Institute for Molecular Medicine Finland (FIMM), HiLIFE, University of Helsinki, Helsinki, Finland	FinnGen Analysis working group	FinnGen Analysis working group
Andrea Ganna	Institute for Molecular Medicine Finland (FIMM), HiLIFE, University of Helsinki, Helsinki, Finland	FinnGen Analysis working group	FinnGen Analysis working group
Vincent Llorens	Institute for Molecular Medicine Finland (FIMM), HiLIFE, University of Helsinki, Helsinki, Finland	FinnGen Analysis working group	FinnGen Analysis working group
Hannele Laivuori	Institute for Molecular Medicine Finland (FIMM), HiLIFE, University of Helsinki, Helsinki, Finland	FinnGen Analysis working group	FinnGen Analysis working group
Mary Pat Reeve	Institute for Molecular Medicine Finland (FIMM), HiLIFE, University of Helsinki, Helsinki, Finland	FinnGen Analysis working group	FinnGen Analysis working group
Henrike Heyne	Institute for Molecular Medicine Finland (FIMM), HiLIFE, University of Helsinki, Helsinki, Finland	FinnGen Analysis working group	FinnGen Analysis working group
Nina Mars	Institute for Molecular Medicine Finland (FIMM), HiLIFE, University of Helsinki, Helsinki, Finland	FinnGen Analysis working group	FinnGen Analysis working group
Joel Rämö	Institute for Molecular Medicine Finland (FIMM), HiLIFE, University of Helsinki, Helsinki, Finland	FinnGen Analysis working group	FinnGen Analysis working group
Hanna Ollila	Institute for Molecular Medicine Finland (FIMM), HiLIFE, University of Helsinki, Helsinki, Finland	FinnGen Analysis working group	FinnGen Analysis working group
Elmo Saarentaus	Institute for Molecular Medicine Finland (FIMM), HiLIFE, University of Helsinki, Helsinki, Finland	FinnGen Analysis working group	FinnGen Analysis working group
Rodos Rodosthenous	Institute for Molecular Medicine Finland (FIMM), HiLIFE, University of Helsinki, Helsinki, Finland	FinnGen Analysis working group	FinnGen Analysis working group
Shabbeer Hassan	Institute for Molecular Medicine Finland (FIMM), HiLIFE, University of Helsinki, Helsinki, Finland	FinnGen Analysis working group	FinnGen Analysis working group
Satu Strausz	Institute for Molecular Medicine Finland (FIMM), HiLIFE, University of Helsinki, Helsinki, Finland	FinnGen Analysis working group	FinnGen Analysis working group
Taru Tukiainen	Institute for Molecular Medicine Finland (FIMM), HiLIFE, University of Helsinki, Helsinki, Finland	FinnGen Analysis working group	FinnGen Analysis working group
Tuula Palotie	University of Helsinki and Hospital District of Helsinki and Uusimaa, Helsinki, Finland	FinnGen Analysis working group	FinnGen Analysis working group
Kimmo Palin	University of Helsinki, Helsinki, Finland	FinnGen Analysis working group	FinnGen Analysis working group
Javier Garcia-Tabuenca	University of Tampere, Tampere, Finland	FinnGen Analysis working group	FinnGen Analysis working group
Harri Siirtola	University of Tampere, Tampere, Finland	FinnGen Analysis working group	FinnGen Analysis working group
Tuomo Kiiskinen	Institute for Molecular Medicine Finland (FIMM), HiLIFE, University of Helsinki, Helsinki, Finland	FinnGen Analysis working group	FinnGen Analysis working group
Jiwoo Lee	Institute for Molecular Medicine Finland (FIMM), HiLIFE, University of Helsinki, Helsinki, Finland; Broad Institute, Cambridge, MA, United States	FinnGen Analysis working group	FinnGen Analysis working group

Kristin Tsuo	Institute for Molecular Medicine Finland (FIMM), HiLIFE, University of Helsinki, Helsinki, Finland; Broad Institute, Cambridge, MA, United States	FinnGen Analysis working group	FinnGen Analysis working group
Amanda Elliott	Institute for Molecular Medicine Finland (FIMM), HiLIFE, University of Helsinki, Helsinki, Finland; Broad Institute, Cambridge, MA, USA and Massachusetts General Hospital, Boston, MA, USA	FinnGen Analysis working group	FinnGen Analysis working group
Kati Kristiansson	THL Biobank / Finnish Institute for Health and Welfare (THL), Helsinki, Finland	FinnGen Analysis working group	FinnGen Analysis working group
Mikko Arvas	Finnish Red Cross Blood Service / Finnish Hematology Registry and Clinical Biobank, Helsinki, Finland	FinnGen Analysis working group	FinnGen Analysis working group
Kati Hyvärinen	Finnish Red Cross Blood Service, Helsinki, Finland	FinnGen Analysis working group	FinnGen Analysis working group
Jarmo Ritari	Finnish Red Cross Blood Service, Helsinki, Finland	FinnGen Analysis working group	FinnGen Analysis working group
Olli Carpén	Helsinki Biobank / Helsinki University and Hospital District of Helsinki and Uusimaa, Helsinki	FinnGen Analysis working group	FinnGen Analysis working group
Johannes Kettunen	Northern Finland Biobank Borealis / University of Oulu / Northern Ostrobothnia Hospital District, Oulu, Finland	FinnGen Analysis working group	FinnGen Analysis working group
Katri Pylkäs	University of Oulu, Oulu, Finland	FinnGen Analysis working group	FinnGen Analysis working group
Eeva Sliz	University of Oulu, Oulu, Finland	FinnGen Analysis working group	FinnGen Analysis working group
Minna Karjalainen	University of Oulu, Oulu, Finland	FinnGen Analysis working group	FinnGen Analysis working group
Tuomo Mantere	Northern Finland Biobank Borealis / University of Oulu / Northern Ostrobothnia Hospital District, Oulu, Finland	FinnGen Analysis working group	FinnGen Analysis working group
Eeva Kangasniemi	Finnish Clinical Biobank Tampere / University of Tampere / Pirkanmaa Hospital District, Tampere, Finland	FinnGen Analysis working group	FinnGen Analysis working group
Sami Heikkinen	University of Eastern Finland, Kuopio, Finland	FinnGen Analysis working group	FinnGen Analysis working group
Arto Mannermaa	Biobank of Eastern Finland / University of Eastern Finland / Northern Savo Hospital District, Kuopio, Finland	FinnGen Analysis working group	FinnGen Analysis working group
Eija Laakkonen	University of Jyväskylä, Jyväskylä, Finland	FinnGen Analysis working group	FinnGen Analysis working group
Nina Pitkänen	Auria Biobank / University of Turku / Hospital District of Southwest Finland, Turku, Finland	FinnGen Analysis working group	FinnGen Analysis working group
Samuel Lessard	Translational Sciences, Sanofi R&D, Framingham, MA, USA	FinnGen Analysis working group	FinnGen Analysis working group
Clément Chatelain	Translational Sciences, Sanofi R&D, Framingham, MA, USA	FinnGen Analysis working group	FinnGen Analysis working group
Perttu Terho	Auria Biobank / University of Turku / Hospital District of Southwest Finland, Turku, Finland	Biobank directors	Biobank directors
Tiina Wahlfors	THL Biobank / Finnish Institute for Health and Welfare (THL), Helsinki, Finland	Biobank directors	Biobank directors
Jukka Partanen	Finnish Red Cross Blood Service / Finnish Hematology Registry and Clinical Biobank, Helsinki, Finland	Biobank directors	Biobank directors
Eero Punkka	Helsinki Biobank / Helsinki University and Hospital District of Helsinki and Uusimaa, Helsinki	Biobank directors	Biobank directors
Raisa Serpi	Northern Finland Biobank Borealis / University of Oulu / Northern Ostrobothnia Hospital District, Oulu, Finland	Biobank directors	Biobank directors
Sanna Siltanen	Finnish Clinical Biobank Tampere / University of Tampere / Pirkanmaa Hospital District, Tampere, Finland	Biobank directors	Biobank directors
Veli-Matti Kosma	Biobank of Eastern Finland / University of Eastern Finland / Northern Savo Hospital District, Kuopio, Finland	Biobank directors	Biobank directors
Teijo Kuopio	Central Finland Biobank / University of Jyväskylä / Central Finland Health Care District, Jyväskylä, Finland	Biobank directors	Biobank directors
Anu Jalanko	Institute for Molecular Medicine Finland (FIMM), HiLIFE, University of Helsinki, Helsinki, Finland	FinnGen Teams	Administration
Huei-Yi Shen	Institute for Molecular Medicine Finland (FIMM), HiLIFE, University of Helsinki, Helsinki, Finland	FinnGen Teams	Administration
Risto Kajanne	Institute for Molecular Medicine Finland (FIMM), HiLIFE, University of Helsinki, Helsinki, Finland	FinnGen Teams	Administration
Mervi Aavikko	Institute for Molecular Medicine Finland (FIMM), HiLIFE, University of Helsinki, Helsinki, Finland	FinnGen Teams	Administration
Henna Palin	Finnish Clinical Biobank Tampere / University of Tampere / Pirkanmaa Hospital District, Tampere, Finland	FinnGen Teams	Administration
Malla-Maria Linna	Helsinki Biobank / Helsinki University and Hospital District of Helsinki and Uusimaa, Helsinki	FinnGen Teams	Administration
Juha Karjalainen	Institute for Molecular Medicine Finland (FIMM), HiLIFE, University of Helsinki, Helsinki, Finland	FinnGen Teams	Analysis
Mitja Kurki	Institute for Molecular Medicine Finland (FIMM), HiLIFE, University of Helsinki, Helsinki, Finland	FinnGen Teams	Analysis
Pietro Della Briotta Parolo	Institute for Molecular Medicine Finland (FIMM), HiLIFE, University of Helsinki, Helsinki, Finland	FinnGen Teams	Analysis
Arto Lehisto	Institute for Molecular Medicine Finland (FIMM), HiLIFE, University of Helsinki, Helsinki, Finland	FinnGen Teams	Analysis
Juha Mehtonen	Institute for Molecular Medicine Finland (FIMM), HiLIFE, University of Helsinki, Helsinki, Finland	FinnGen Teams	Analysis
Wei Zhou	Broad Institute, Cambridge, MA, United States	FinnGen Teams	Analysis
Masahiro Kanai	Broad Institute, Cambridge, MA, United States	FinnGen Teams	Analysis
Mutaamba Maasha	Broad Institute, Cambridge, MA, United States	FinnGen Teams	Analysis
Zhili Zheng	Broad Institute, Cambridge, MA, United States	FinnGen Teams	Analysis
Hannele Laivuori	Institute for Molecular Medicine Finland (FIMM), HiLIFE, University of Helsinki, Helsinki, Finland	FinnGen Teams	Clinical Endpoint Development
Aki Havulinna	Institute for Molecular Medicine Finland (FIMM), HiLIFE, University of Helsinki, Helsinki, Finland	FinnGen Teams	Clinical Endpoint Development

Susanna Lemmelä	Institute for Molecular Medicine Finland (FIMM), HiLIFE, University of Helsinki, Helsinki, Finland	FinnGen Teams	Clinical Endpoint Development
Tuomo Kiiskinen	Institute for Molecular Medicine Finland (FIMM), HiLIFE, University of Helsinki, Helsinki, Finland	FinnGen Teams	Clinical Endpoint Development
Elisa Lahtela	Institute for Molecular Medicine Finland (FIMM), HiLIFE, University of Helsinki, Helsinki, Finland	FinnGen Teams	Clinical Endpoint Development
Mari Kaunisto	Institute for Molecular Medicine Finland (FIMM), HiLIFE, University of Helsinki, Helsinki, Finland	FinnGen Teams	Communication
Elina Kilpeläinen	Institute for Molecular Medicine Finland (FIMM), HiLIFE, University of Helsinki, Helsinki, Finland	FinnGen Teams	E-Science
Timo P. Sipilä	Institute for Molecular Medicine Finland (FIMM), HiLIFE, University of Helsinki, Helsinki, Finland	FinnGen Teams	E-Science
Oluwaseun Alexander Dad	Institute for Molecular Medicine Finland (FIMM), HiLIFE, University of Helsinki, Helsinki, Finland	FinnGen Teams	E-Science
Awaisa Ghazal	Institute for Molecular Medicine Finland (FIMM), HiLIFE, University of Helsinki, Helsinki, Finland	FinnGen Teams	E-Science
Anastasia Kytölä	Institute for Molecular Medicine Finland (FIMM), HiLIFE, University of Helsinki, Helsinki, Finland	FinnGen Teams	E-Science
Sanni Ruotsalainen	Institute for Molecular Medicine Finland (FIMM), HiLIFE, University of Helsinki, Helsinki, Finland	FinnGen Teams	E-Science
Rigbe Weldatsadik	Institute for Molecular Medicine Finland (FIMM), HiLIFE, University of Helsinki, Helsinki, Finland	FinnGen Teams	E-Science
Kati Donner	Institute for Molecular Medicine Finland (FIMM), HiLIFE, University of Helsinki, Helsinki, Finland	FinnGen Teams	Genotyping
Timo P. Sipilä	Institute for Molecular Medicine Finland (FIMM), HiLIFE, University of Helsinki, Helsinki, Finland	FinnGen Teams	Genotyping
Anu Loukola	Helsinki Biobank / Helsinki University and Hospital District of Helsinki and Uusimaa, Helsinki	FinnGen Teams	Sample Collection Coordination
Päivi Laiho	THL Biobank / Finnish Institute for Health and Welfare (THL), Helsinki, Finland	FinnGen Teams	Sample Logistics
Tuuli Sistonen	THL Biobank / Finnish Institute for Health and Welfare (THL), Helsinki, Finland	FinnGen Teams	Sample Logistics
Essi Kaiharju	THL Biobank / Finnish Institute for Health and Welfare (THL), Helsinki, Finland	FinnGen Teams	Sample Logistics
Markku Laukkanen	THL Biobank / Finnish Institute for Health and Welfare (THL), Helsinki, Finland	FinnGen Teams	Sample Logistics
Elina Järvensivu	THL Biobank / Finnish Institute for Health and Welfare (THL), Helsinki, Finland	FinnGen Teams	Sample Logistics
Sini Lähteenmäki	THL Biobank / Finnish Institute for Health and Welfare (THL), Helsinki, Finland	FinnGen Teams	Sample Logistics
Lotta Männikkö	THL Biobank / Finnish Institute for Health and Welfare (THL), Helsinki, Finland	FinnGen Teams	Sample Logistics
Regis Wong	THL Biobank / Finnish Institute for Health and Welfare (THL), Helsinki, Finland	FinnGen Teams	Sample Logistics
Auli Toivola	THL Biobank / Finnish Institute for Health and Welfare (THL), Helsinki, Finland	FinnGen Teams	Sample Logistics
Minna Brunfeldt	THL Biobank / Finnish Institute for Health and Welfare (THL), Helsinki, Finland	FinnGen Teams	Registry Data Operations
Hannele Mattsson	THL Biobank / Finnish Institute for Health and Welfare (THL), Helsinki, Finland	FinnGen Teams	Registry Data Operations
Kati Kristiansson	THL Biobank / Finnish Institute for Health and Welfare (THL), Helsinki, Finland	FinnGen Teams	Registry Data Operations
Susanna Lemmelä	Institute for Molecular Medicine Finland (FIMM), HiLIFE, University of Helsinki, Helsinki, Finland	FinnGen Teams	Registry Data Operations
Sami Koskelainen	THL Biobank / Finnish Institute for Health and Welfare (THL), Helsinki, Finland	FinnGen Teams	Registry Data Operations
Tero Hiekkalinna	THL Biobank / Finnish Institute for Health and Welfare (THL), Helsinki, Finland	FinnGen Teams	Registry Data Operations
Teemu Paajanen	THL Biobank / Finnish Institute for Health and Welfare (THL), Helsinki, Finland	FinnGen Teams	Registry Data Operations
Priit Palta	Institute for Molecular Medicine Finland (FIMM), HiLIFE, University of Helsinki, Helsinki, Finland	FinnGen Teams	Sequencing Informatics
Kalle Pärn	Institute for Molecular Medicine Finland (FIMM), HiLIFE, University of Helsinki, Helsinki, Finland	FinnGen Teams	Sequencing Informatics
Mart Kals	Institute for Molecular Medicine Finland (FIMM), HiLIFE, University of Helsinki, Helsinki, Finland	FinnGen Teams	Sequencing Informatics
Shuang Luo	Institute for Molecular Medicine Finland (FIMM), HiLIFE, University of Helsinki, Helsinki, Finland	FinnGen Teams	Sequencing Informatics
Tarja Laitinen	Pirkanmaa Hospital District, Tampere, Finland	FinnGen Teams	Trajectory
Mary Pat Reeve	Institute for Molecular Medicine Finland (FIMM), HiLIFE, University of Helsinki, Helsinki, Finland	FinnGen Teams	Trajectory
Shanmukha Sampath Padsam	Institute for Molecular Medicine Finland (FIMM), HiLIFE, University of Helsinki, Helsinki, Finland	FinnGen Teams	Trajectory
Marianna Niemi	University of Tampere, Tampere, Finland	FinnGen Teams	Trajectory
Harri Siirtola	University of Tampere, Tampere, Finland	FinnGen Teams	Trajectory
Javier Gracia-Tabuenca	University of Tampere, Tampere, Finland	FinnGen Teams	Trajectory
Mika Helminen	University of Tampere, Tampere, Finland	FinnGen Teams	Trajectory
Tiina Luukkaala	University of Tampere, Tampere, Finland	FinnGen Teams	Trajectory
Iida Vähätalo	University of Tampere, Tampere, Finland	FinnGen Teams	Trajectory
Jyrki Tammerluoto	Institute for Molecular Medicine Finland (FIMM), HiLIFE, University of Helsinki, Helsinki, Finland	FinnGen Teams	Data protection officer

Marco Hautalahti	Finnish Biobank Cooperative - FINBB	FinnGen Teams	FINBB - Finnish biobank cooperative
Johanna Mäkelä	Finnish Biobank Cooperative - FINBB	FinnGen Teams	FINBB - Finnish biobank cooperative
Sarah Smith	Finnish Biobank Cooperative - FINBB	FinnGen Teams	FINBB - Finnish biobank cooperative
Tom Southerington	Finnish Biobank Cooperative - FINBB	FinnGen Teams	FINBB - Finnish biobank cooperative
Petri Lehto	Finnish Biobank Cooperative - FINBB	FinnGen Teams	FINBB - Finnish biobank cooperative
