# Supplementary material for: Genetic analyses implicate complex links between adult testosterone levels and health and disease
Source: Commun Med (Lond). 2023 Jan 18;3:4. doi: 10.1038/s43856-022-00226-0 (PMC9849476; doi:10.1038/s43856-022-00226-0)
Supplement: Supplementary file 16 — Description of Additional Supplementary Files [file 43856_2022_226_MOESM16_ESM.pdf]

Description of Additional Supplementary Files

File Name: Supplementary Data 1

Description: Lead SNPs from the GWAS loci for testosterone, SHBG, FAI and free testosterone, with annotations.

File Name: Supplementary Data 2

Description: GWAS summaries and genetic connections between the studied testosterone traits based on the UK Biobank data.

File Name: Supplementary Data 3

Description: Results from genetic pathway analyses implemented in FUMA.

File Name: Supplementary Data 4

Description: Sharing of total testosterone, SHBG, FAI and free testosterone loci between males and females based on gwas-pw analyses.

File Name: Supplementary Data 5

Description: Properties of the sex-shared loci for total testosterone and free testosterone.

File Name: Supplementary Data 6

Description: Characteristics of the Young Finns Cohort and results from the replication analyses for T and related traits.

File Name: Supplementary Data 7

Description: Results from PGS analyses in FinnGen.

File Name: Supplementary Data 8

Description: Results from latent causal variable (LCV) and Mendelian Randomisation (MR) analyses in FinnGen.

File Name: Supplementary Data 9

Description: Results from cross-sex PGS analyses in FinnGen.

File Name: Supplementary Data 10

Description: Highlights from cross-sex analyses in FinnGen.

File Name: Supplementary Data 11

Description: Genetic correlation (rg) of T, SHBG, FAI and free T over 44 selected traits from published GWAS.

File Name: Supplementary Data 12

Description: Results from latent causal variable (LCV) and Mendelian Randomisation (MR) analyses over 44 selected traits from published GWAS.

File Name: Supplementary Data 13

Description: Genetic correlation (rg) results between sex-specific GWAS in the UK Biobank.
