# Supplementary material for: Genetic analyses implicate complex links between adult testosterone levels and health and disease
Source: Commun Med (Lond). 2023 Jan 18;3:4. doi: 10.1038/s43856-022-00226-0 (PMC9849476; doi:10.1038/s43856-022-00226-0)
Supplement: Supplementary file 17 — Reporting Summary [file 43856_2022_226_MOESM17_ESM.pdf]

nature research

Corresponding author(s): Taru Tukiainen

Last updated by author(s): Dec 7, 2022

Reporting Summary

Nature Research wishes to improve the reproducibility of the work that we publish. This form provides structure for consistency and transparency in reporting. For further information on Nature Research policies, see our Editorial Policies and the Editorial Policy Checklist.

Statistics

For all statistical analyses, confirm that the following items are present in the figure legend, table legend, main text, or Methods section.

n/a	Confirmed	
☐	☒	The exact sample size (n) for each experimental group/condition, given as a discrete number and unit of measurement
☐	☒	A statement on whether measurements were taken from distinct samples or whether the same sample was measured repeatedly
☐	☒	The statistical test(s) used AND whether they are one- or two-sided<br>*Only common tests should be described solely by name; describe more complex techniques in the Methods section.*
☐	☒	A description of all covariates tested
☐	☒	A description of any assumptions or corrections, such as tests of normality and adjustment for multiple comparisons
☐	☒	A full description of the statistical parameters including central tendency (e.g. means) or other basic estimates (e.g. regression coefficient) AND variation (e.g. standard deviation) or associated estimates of uncertainty (e.g. confidence intervals)
☐	☒	For null hypothesis testing, the test statistic (e.g. F, t, r) with confidence intervals, effect sizes, degrees of freedom and P value noted<br>*Give P values as exact values whenever suitable.*
☐	☒	For Bayesian analysis, information on the choice of priors and Markov chain Monte Carlo settings
☐	☒	For hierarchical and complex designs, identification of the appropriate level for tests and full reporting of outcomes
☐	☒	Estimates of effect sizes (e.g. Cohen's d, Pearson's r), indicating how they were calculated

Our web collection on statistics for biologists contains articles on many of the points above.

Software and code

Policy information about availability of computer code

Data collection	The full genotyping and imputation protocol for FinnGen is described at https://doi.org/10.17504/protocols.io.nmndc5e.
Data analysis	The GWAS analyses were performed using BOLT-LMM (v2.3.2). Tissue and gene set enrichment analyses were carried out with SNP2GENE and GENE2FUNC implemented in FUMA using default settings. LDstore2 (v2.0b) was used to calculate LD in YFS. To construct PGSs in UKBB, YFS and FinnGen we applied weights from LDpred, using the infinitesimal model in Plink 2.0. SNP-based heritability and genetic correlations were estimated using linkage disequilibrium score regression (LDSC). Cox proportional hazards models were used for estimating hazard ratios (HRs) and 95% CIs in FinnGen. The proportionality assumption for Cox models was assessed with Schoenfeld residuals and log-log plots. Latent causal variable (LCV), MR-Egger, multivariable MR-Egger and Inverse Variance Weighed (IVW) were used for Mendelian Randomisation. The MR analyses were run using TwoSampleMR (v0.5.2) and MendelianRandomization (v0.5.0) R packages using R 4.0.2.

For manuscripts utilizing custom algorithms or software that are central to the research but not yet described in published literature, software must be made available to editors and reviewers. We strongly encourage code deposition in a community repository (e.g. GitHub). See the Nature Research guidelines for submitting code & software for further information.

Data

Policy information about availability of data

All manuscripts must include a data availability statement. This statement should provide the following information, where applicable:

- Accession codes, unique identifiers, or web links for publicly available datasets
- A list of figures that have associated raw data
- A description of any restrictions on data availability

The source data for Figures is available in Supplementary Data: for Figure 1 – Supplementary Data 2; Figure 2 – Supplementary Data 7; Figure 3 – Supplementary Data 8; Figure 4 – Supplementary Data 9; Figure 5 – Supplementary Data 10&11. Full genetic and clinical data from FinnGen and the UK Biobank are available for

researchers by application, https://www.finngen.fi/en/access_results and https://www.ukbiobank.ac.uk/. For YFS see https://youngfinnsstudy.utu.fi/index.html. GWAS summary statistics for total T, SHBG, FAI and free T based on the UK Biobank data will be available from the GWAS catalogue (https://www.ebi.ac.uk/gwas) with submission ID GCP000476. Data from publicly available GWASs can be downloaded from the source repositories with references listed in Supplementary Data 11.

Field-specific reporting

Please select the one below that is the best fit for your research. If you are not sure, read the appropriate sections before making your selection.

☒ Life sciences ☐ Behavioural & social sciences ☐ Ecological, evolutionary & environmental sciences

For a reference copy of the document with all sections, see nature.com/documents/nr-reporting-summary-flat.pdf

Life sciences study design

All studies must disclose on these points even when the disclosure is negative.

Sample size	The study includes data from ~630,000 biobank participants. No sample size calculations were performed given the nature of the study.
Data exclusions	We restricted our study to encompass 408,186 individuals from the white British subset. For GWASs, we removed outliers for genotype heterozygosity and missingness, as well as samples with sex chromosome aneuploidies, mismatches between reported and inferred sex, and samples that UK Biobank did not use in relatedness calculations, and withdrawn participants. Subjects with residuals values of +- 5SD from the mean were excluded from the UK Biobank analyses. Relatives, pregnant women and outliers for biomarker measurements (+/- 5SD) were excluded from the YFS.
Replication	Predictive ability of the PGS was tested in an independent cohort (the Young Finns Study, YFS). Publicly available GWAS summary statistics were used to replicate and extend the genetic findings from FinnGen.
Randomization	FinnGen participants were allocated case/control status based on national health registry data. For construction of the sex-specific PGS and replication in the YFS, first 10 genetic PCs, age and BMI were used as covariates.
Blinding	Blinding was not necessary given the nature of the study.

Reporting for specific materials, systems and methods

We require information from authors about some types of materials, experimental systems and methods used in many studies. Here, indicate whether each material, system or method listed is relevant to your study. If you are not sure if a list item applies to your research, read the appropriate section before selecting a response.

Materials & experimental systems

n/a	Involved in the study	
☒	☐	Antibodies
☒	☐	Eukaryotic cell lines
☒	☐	Palaeontology and archaeology
☒	☐	Animals and other organisms
☐	☒	Human research participants
☒	☐	Clinical data
☒	☐	Dual use research of concern

Methods

n/a	Involved in the study	
☒	☐	ChIP-seq
☒	☐	Flow cytometry
☒	☐	MRI-based neuroimaging

Human research participants

Policy information about studies involving human research participants

Population characteristics	The detailed characteristics of the three biobank cohorts are available at www.ukbiobank.ac.uk, www.finngen.fi and at https://youngfinnsstudy.utu.fi/. The UK Biobank is a prospective cohort with genetic, physical and health data from ~500,000 individuals, aged betwen 39 and 71, collected across the United Kingdom between 2006-2010. FinnGen is a public-private partnership project combining genotype data and digital health record data from Finnish health registries, comprised of Finnish prospective epidemiological and disease-based cohorts and voluntary biobank samples, launched in 2017. For this study, we utilized FinnGen R5, containing 217,464 participants. The Cardiovascular Risk in Young Finns Study (YFS) was designed as a follow-up study into cardiovascular risk from childhood to adulthood. The first cross-sectional survey for YFS was conducted in 1980. Total sample size was 4,320 boys and girls, and the subjects were randomly chosen from the national register.
Recruitment	Study included data from participants of the UK Biobank, FinnGen and the Young Finns Study consortiums. The recruitement procedures for these biobanks are available through the official websites for these studies.

Ethics oversight

Research was performed in accordance with the declaration of Helsinki and approved by the local ethics committees. Informed consent was obtained from all participants. This study was run under UK Biobank application number 22627.

Note that full information on the approval of the study protocol must also be provided in the manuscript.
